# Evaluation of the anti-SARS-CoV-2 properties of essential oils and aromatic extracts

**DOI:** 10.1038/s41598-022-18676-w

**Published:** 2022-08-20

**Authors:** Daniel Jan Strub, Michał Talma, Maria Strub, Wioletta Rut, Mikolaj Zmudzinski, Władysław Brud, Johan Neyts, Laura Vangeel, Linlin Zhang, Xinyuanyuan Sun, Zongyang Lv, Digant Nayak, Shaun K. Olsen, Rolf Hilgenfeld, Dirk Jochmans, Marcin Drąg

**Affiliations:** 1 Department of Chemical Biology and Bioimaging, Wrocław University of Science and Technology, Wyb. Wyspiańskiego 27, 50-370 Wrocław, Poland; 2 Liquid Technologies sp. z o.o, Gdańska 13, 50-344 Wrocław, Poland; 3 Department of Bioorganic Chemistry, Wrocław University of Science and Technology, Wyb. Wyspiańskiego 27, 50-370 Wrocław, Poland; 4 Scientific Committee of the International Federation of Essential Oils and Aroma Trades (IFEAT), c/o TC Group, Level 1, Devonshire House, One Mayfair Place, London, W1J 8AJ UK; 5 Laboratory of Virology and Chemotherapy, Department of Microbiology, Immunology and Transplantation, Rega Institute, KU Leuven, Leuven, Belgium; 6 Institute of Molecular Medicine, University of Lübeck, Ratzeburger Allee 160, 23562 Lübeck, Germany; 7 Department of Biochemistry and Molecular Biology and Hollings Cancer Center, Medical University of South Carolina, Charleston, SC 29425 USA; 8 Department of Biochemistry and Structural Biology, University of Texas Health Science Center at San Antonio, San Antonio, TX 78229 USA; 9 German Center for Infection Research (DZIF), Hamburg-Lübeck-Borstel-Riems Site, University of Lübeck, 23562 Lübeck, Germany

**Keywords:** Proteases, SARS-CoV-2

## Abstract

Essential oils and aromatic extracts (oleoresins, absolutes, concretes, resinoids) are often used as food flavorings and constituents of fragrance compositions. The flavor and fragrance industry observed significant growth in the sales of some natural materials during the COVID-19 outbreak. Some companies worldwide are making false claims regarding the effectiveness of their essential oils or blends (or indirectly point toward this conclusion) against coronaviruses, even though the available data on the activity of plant materials against highly pathogenic human coronaviruses are very scarce. Our exploratory study aimed to develop pioneering knowledge and provide the first experimental results on the inhibitory properties of hundreds of flavor and fragrance materials against SARS-CoV-2 main and papain-like proteases and the antiviral potential of the most active protease inhibitors. As essential oils are volatile products, they could provide an interesting therapeutic strategy for subsidiary inhalation in the long term.

## Introduction

Essential oils (EOs) are plant-derived products and often consist of dozens to hundreds of volatile compounds. They are produced only by physical methods such as mechanical pressing or distillation (hydrodistillation, steam distillation)^1^. Aromatic extracts (AEs), conversely, are obtained by extraction with organic solvents by classical solid–liquid extraction, solid–solid extraction (enfleurage), or modern equipment (supercritical fluid and microwave extractors). Essential oils and aromatic extracts are often used as food flavorings, perfuming agents, pharmaceuticals, and the former for aromatherapy. The complexity of EO and AE matrices is an undesirable trait in drug discovery, but they are useful in some areas of medical therapy and can be a promising source of new drugs^2^. Essential oils and aromatic extracts are not considered highly effective antiviral agents, and in the flavor and fragrance scientific community, interest has shifted more toward the assessment of the antimicrobial properties of these plant-derived products. Studies regarding the antiviral properties of volatile natural materials mostly concern in vitro evaluations^3^; in vivo testing of these materials is very rare^4^, and in vivo human data are virtually nonexistent.

The flavor and fragrance industry observed significant growth in the sales of some natural materials during the coronavirus disease-2019 (COVID-19) outbreak. This growth was attributed in part to the aromatherapeutic properties of the selected products but also in part to fraudulent marketing. Some companies worldwide are making false claims regarding the effectiveness of their essential oils (or indirectly point toward this conclusion) against coronaviruses and (in the same marketing message) their ability to boost the immune system during respiratory illnesses.

Coronaviruses (fam. *Coronaviridae*) are crown-like enveloped viruses comprising single-stranded, positive-sense RNA. Host targets for coronaviruses are animals and humans. Before the current outbreak, HCoV-229E, HCoV-NL63, HCoV-OC43, HCoV-HKU1, SARS-CoV, and MERS-CoV were known to be able to infect humans^5^. The first four are the cause of (in most cases) mild illnesses (common colds), whereas infections with SARS-CoV and MERS-CoV can develop into a severe respiratory disease that could be fatal. Severe acute respiratory syndrome coronavirus 2 (SARS-CoV-2), which is responsible for the current outbreak of the disease (COVID-19), is the seventh coronavirus known to infect humans^6^. Due to international travel and human–human interactions, the virus spread rapidly, which has resulted in more than 500 million infections and over 6 000 000 deaths (according to Worldometers Info, accessed June 30, 2022). The world scientific community and pharmaceutical industry are devoted to finding an effective therapy for COVID-19. In addition to the existing protective vaccinations against COVID-19, targeted drug therapy plays a very important role; however, despite many efforts, it has not been sufficiently developed thus far. A preliminary study showed that drugs from many groups, such as antibacterials, antiprotozoals, immunomodulators, angiotensin II receptor blockers, bradykinin B2 receptor antagonists, corticosteroids, anthelmintics, H2 blockers, and anticoagulants, are considered potential therapeutics in COVID-19 treatment^7^.

Two of the very well characterized and promising drug targets are the main protease (M^pro^, 3CL^pro^) and the papain-like protease (PL^pro^), which play key roles in viral replication and transcription^8,9^. SARS-CoV-2 PL^pro^ also exhibits deubiquitinating and deISGylating activities, which are implicated in suppressing host innate immune responses^9,10^. Therefore, research efforts are focused on the rapid development of SARS-CoV-2 M^pro^ and PL^pro^ inhibitors as drug candidates. Several approaches have been utilized in coronaviral protease inhibitor-drug discovery, including structure-based drug design^8,11–15^, high-throughput (HT) screening of structurally diverse compound libraries^16–18^, in silico studies^19–21^, and drug repurposing^22–25^. Despite substantial efforts, only one compound (PF-07321332, brand name Paxlovid) has been approved by the US Food and Drug Administration (FDA) for the treatment of mild-to-moderate coronavirus disease^26^, and one (S-217622) is in late clinical trials (NCT05305547)^21^. Both compounds act as SARS-CoV-2 M^pro^ inhibitors and are administered orally. Unlike M^pro^, few SARS-CoV-2 PL^pro^ inhibitors with experimentally proven efficacy have been reported^17,27–29^. Due to the restricted binding pockets at the P1 and P2 substrate-binding sites and the preference for relatively large ubiquitin-like proteins over short peptide substrates, inhibitor-drug design toward SARS-CoV-2 PL^pro^ is much more challenging^10,30^.

In the present study, we wanted to address the question of whether common flavor and fragrance materials are inhibitors or have the potential to be a source for effective inhibitors of the main and papain-like proteases of SARS-CoV-2 and whether these inhibitors exhibit in vitro antiviral properties. As essential oils are volatile products, they could provide an interesting therapeutic strategy for subsidiary inhalation in the long term. To the best of our knowledge, this is the first experimental study^31^ regarding the evaluation of the potential of flavors and fragrances as a part of the search for recent anti-coronaviral strategies.

## Materials and methods

The molecular modeling studies were prepared with the protocol of our earlier studies^32^. Crystal structures of SARS-CoV-2 M^pro^ (PDB ID: 6XBH^33^) and SARS-CoV-2 PL^pro^ (PDB ID: 6WX4^10^) were obtained from the Research Collaboratory for Structural Bioinformatics Protein Data Bank (RCSB-PDB). Waters, ligands, and ions were deleted, and the proteins were protonated at the experimental pH and optimized^34^. Before docking, the structures of the inhibitors were protonated in the experimental pH typical for each enzyme and optimized by LigPrep^35^. The Induced Fit Docking protocol was used for molecular docking studies. The site of the enclosing box was set at 20 Å on the centroid of the catalytic cysteines^36^. The VSGB (variable-dielectric generalized Born) model, which incorporates residue-dependent effects, was used. The solvent was water. The active center amino acids were optimized within 5.0 Å of ligand poses, and Glide redocking was carried out with the XP (extra precision) algorithm. The top three poses for the ligand were saved. The last steps, rePrime refinement, and MM-GBSA (molecular mechanics-generalized Born surface area) calculations were performed to calculate the Gibbs free energies with protein flexibility, and the distance from the ligand was also set to 5.0 Å. The one with the lowest free binding energy was considered for publication. The protocol and all the parameters were the same for the second docking, but the ligands were forced to interact with the catalytic cysteine (Cys145 or Cys111) during the IFD.

### Reagents

#### Essential oils and related products

All samples of investigated plant volatile products were generously donated by members of the industry – manufacturers of the products at the best possible quality. Nomenclature of the products according to specifications.

They were:

**A. Fakhry & Co.**

Essential oils of bitter orange (fruit) distilled, petitgrain bigarade *sur fleurs*, cabbage rose, carrot seed, celery seed, celery leaf, chamomile blue, cinnamon basil, tropical basil, sweet basil, lemon basil, clary sage, coriander seed, coriander leaf, dill leaf, jasmine, anise seed, cumin, lantana, leek, fennel bitter, fennel sweet, neroli, neroli BdN, parsley seed, parsley leaf, petitgrain bigarade, petitgrain key lime, petitgrain mandarin, winter savory, summer savory, tagetes, geranium, petitgrain mandarin, bitter orange distilled, rosemary, caraway, aniseed, spearmint, sweet marjoram, mature bitter orange (“red”) expressed, immature bitter orange (“green”) expressed, onion, and garlic, absolutes of artichoke, cabbage rose, cabbage rose leaves, calendula, carnation, carrot leaf, cassie, *Rosa damascena*, bitter orange blossom hydrolate, honeysuckle, jasmine, mango leaf, nasturtium, nettle, olive leaf, rocket, spinach, strawberry leaf, tomato leaf, tropical basil, sweet basil, lemon basil, tropical basil hydrolate, petitgrain bigarade, tagetes, geranium, carrot seed, coriander leaves, fenugreek, geranium hydrolate, rosemary, caraway, sweet marjoram, blue chamomile, cumin, orange flower water, violet leaves, and clary sage, concretes of cabbage rose, jasmine, lemon basil, sweet basil, tropical basil, geranium, thyme, blue chamomile, cumin, violet leaves, nettle, calendula, artichoke, nasturtium, mango leaves, cassie, strawberry leaves, olive leaves, clary sage, carnation, rocket, and carrot leaves, and hydroalcoholic extract of licorice.

**Albert Vieille**

Essential oils of traditional and complete cistus and absolutes of cistus, cistus SEV, and tonka bean.

**Amigo & Arditi**

Essential oils of cabreuva red, guaiac wood, citronella organic, and petitgrain bitter orange.

**Ashna Samai**

Essential oils of Irish lace, spiked pepper, copal, ishpingo, and palo santo.

**Berje Inc.**

Essential oils of angelica root, angelica seed, anise (*Pimpinella anisum*), *Artemisia afra*, rosewood, buchu leaf betulina, buchu leaf crenulata, calamus, cascarilla bark, catnip, coffee, cognac white, cubeb, dill seed, Eucalyptus smithii, fir needle China, lavandin abrialis, lovage leaf, lovage root, mandarin red, milfoil, *Ocotea cymbarum*, parsley leaf, *Rosa damascena* Bulgaria, *Rosa damascena* Turkey, peppermint Yakima, *Pinus pumilio*, perilla, sassafras, savory, siam wood, tagetes, valerian root, and wormwood European, and fir balsam, absolutes of spruce, *Rosa damascena* Morocco and Bulgaria, and orris root concrete, and gums of labdanum and storax.

**Bordas S.A.**

Essential oils of labdacistus, rue, bitter fennel, clementine, cypriol, laurel, onion, tarragon, tangerine, petitgrain lemon, petitgrain mandarin, vetiver Java, ylang-ylang 2nd, citronella Java type, orange CP Valencia, and thyme red, absolutes of labdanum, jasmine, spike lavender, thyme red, and thyme gray, and oleoresins of cumin and turmeric.

**BoreA Canada**

Essential oils of black spruce twigs and leaves, bark, and wood, jack pine twigs and leaves, and wood, balsam poplar, balsam fir bark, hops, larch tamarack, white spruce, and Labrador tea.

**Brüder Unterweger**

Essential oils of black pine, maritime pine, silver fir cones, hops, hyssop, yarrow blue 3% and 15% chamazulene, and Venice turpentine I.

**Christodoulou Bros S.A.**

Essential oils of bitter orange, lemon, mandarin, and white grapefruit.

**Citrus and Allied Essences Ltd.**

Essential oils of fir needle Siberian, ginger China, lemon California type CP Extra, lemon Argentina Tucuman CP, orange Valencia CP, orange Brazil CP, orange Brazil fivefold, corn mint India (partially dementholized, *Mentha arvensis*), tangerine Dancy, and geranium China.

**D.V. Deo Industries**

Essential oils of palmarosa, cardamom, gingergrass vetiver, cypriol, angelica root, spikenard, ginger, betel leaf, lemongrass, galangal root, chaulmoogra, and cedarwood.

**Dutjahn Sandalwood Oils**.

*Santalum spicatum* essential oil.

**Essential Oils and Herbs**

Melissa and yarrow essential oils.

**Essential Oils of Tasmania**

Essential oils of bitter fennel, coastal tea tree, fennel (70% fenchone), kunzea, lavender, parsley herb, parsley seed, peppermint, rosemary, big badja gum, smoky tea tree, and southern Rosalina, absolutes of Boronia, Boronia leaves, and Tasmanian pepperberry, and Tasmanian pepperberry extract.

**Eucaforest**

Essential oils of rose geranium, tea tree, lemon-scented tea tree, *Eucalyptus smithii*, *Eucalyptus radiata*, and tagete.

**Hierbas Patagónicas S.R. L**

Essential oils of fabiana, Douglas fir, paramela, and *Pinus ponderosa*.

**Jacarandas International**

Essential oils of psiadia, ylang-ylang I, II, III, and complete, clove bud and leaf, ginger fresh, black pepper, *Helichrysum gymnocephalum*, *Helichrysum bracteiferum, Eucalyptus citriodora*, geranium, niaouli, and ravintsara.

**Kallin Ltd.**

Essential oils of citronella China, garlic China, geranium China, geranium Egypt, ginger China, neroli Morocco, peppermint arvensis China, petitgrain bigarade, sandalwood India, shiu (Ho wood) China, vetiver Bourbon, and ylang-ylang, absolutes of coffee, fenugreek, jasmine India, orris, and orange flower, and ginger China and pink pepper CO_2_ extracts.

**Lebermuth**

Essential oils of allspice, blood orange, catnip, cedarwood Himalayan, *Eucalyptus polybractea*, goldenrod, grapefruit red, *Lavandula stoechas*, mandarin red, Peru balsam, *Rosa damascena*, black spruce, tea tree, ylang-ylang I, and ylang-ylang II, and neem oil.

**Les Arômes du Maroc**

Mastic and neroli essential oils, orange flower water absolute, orange flower and bran concretes, and orris germanica butter.

**Lluch Essence**

Essential oils of cedarleaf, cedarwood Texas, citronella Java type, geranium Egypt, ginger China, ginger Nigeria, gurjun balsam, lavender fresh Bulgarian, lemon Spain, sweet orange CP Valencia, sweet orange CP Brazil, pennyroyal, peppermint Arvensis, thuja, thyme red, star aniseed, cassie, citrate, cognac green, coriander seed, cumin “ex-distilled”, dill leaf, *Eucalyptus radiata*, lemongrass, key lime distilled type Mexico, key lime expressed type Mexico, mandarin green, myrtle, nutmeg, parsley seed, patchouli molecular distillation, patchouli super dark, pimento berry, pimento leaf, pine needle, scotch pine, ravintsara, sweet basil, tropical basil, verbena, ylang-ylang I, and ylang-ylang III and oleoresins of basil, capsicum 1,000,000 SHU, paprika 40,000 SHU, paprika 60,000 SHU, paprika 80,000 SHU, pepper black 40/20, and thyme red.

**Mane Kancor Ingredients Private Limited**

Essential oils of carrot seed, ajowan, black pepper CO_2_, capsicum CO_2_, ginger CO_2_, cumin seed, black cumin, celery seed, fennel, and mace and tuberose absolute and concrete.

**Metainy**

Cotula and neroli essential oils.

**Nelixia**

Cardamom essential oil, storax gum and raw storax gums, and peru and raw peru balsams.

**New Zealand Mānuka Group**

Essential oils of mānuka (MBTK 5 +), mānuka (MBTK 20 +), mānuka (MBTK 25 +), and kanuka.

**Payan Bertrand S.A.**

Essential oils of galbanum, myrrh, olibanum, opoponax, and storax, oakmoss absolute, and resinoids of galbanum, myrrh, olibanum, opoponax, storax, benzoin Siam, and benzoin Sumatra.

**Plant’s Power**

Essential oils of magnolia flower, goldenrod, catnip, rhododendron, fir balsam needle, Labrador tea, sweet fern, black spruce, and yuzu.

**Robertet S.A.**

Bran, blackcurrant buds, and broom absolutes.

**Ultra International**

Essential oils of angelica root, artemisia, *Artemisia taurica*, blood orange, blue cypress, buchu leaf, buddha wood, coriander herb, *Eucalyptus kochii*, hinoki, kumquat, kunzea, lemon myrtle, and Rosalina and CO_2_ extracts of juniper berry, star anise, turmeric, vetiver, black seed, cardamom green, coffee arabica, coffee robusta, and ginger.

**Van Aroma**

Essential oils of clove stem 85% MD, clove stem 85% dark, and cajeput and CO_2_ extracts of cocoa butter, black pepper, clove bud, turmeric, cubeb, cassia bark, ginger, and benzoin crystals.

**Vessel Essential Oils**

Essential oils of lavender organic, oregano liquid fire, oregano organic, lavandin organic, peppermint organic, sweet orange organic, lemon organic, mandarin organic, pine organic, sea fennel organic, melissa organic, helichrysum organic, thyme organic, laurel organic, sage organic, rosemary organic, white fir organic, blue chamomile organic, mastic organic, rose organic, wild carrot seed organic, sweet marjoram organic, cypress organic, geranium organic, clary sage organic, yarrow organic, sweet fennel organic, juniper berry organic, anise organic, dill organic, cistus organic, Roman chamomile, spearmint, grapefruit organic, coriander seed oil, petitgrain organic, basil organic ct linalool, inula, frankinscense Somalia, Emerald frankincense, frankincense Ethiopia, frankincense India, and frankincense Oman.

The remaining samples of essential oils and other aromatic materials were purchased from PerfumersWorld Ltd. Essential oils were named according to the ISO 4720:2018 norm—*Essential oils—Nomenclature* where possible.

#### Enzymes

The expression of recombinant enzymes SARS-CoV-2 M^pro^ and PL^pro^ is described in our previous works ^8,10^.

#### ACC-labeled substrates

The synthesis of ACC-labeled substrate for M^pro^ (Ac-Abu-Tle-Leu-Gln-ACC) and PL^pro^ (Ac-Leu-Arg-Gly-Gly-ACC) is described in our previous works ^10,37^.

### Inhibitor screening

Experimental conditions (concentration of enzyme and substrate, time) were tailored to obtain optimal enzyme activity during measurement like in our previous communications: PL^pro^^10^ and M^pro^^38^. Control experiments were carried out in the absence of the inhibitor (100% activity), enzyme or substrate. SARS-CoV-2 M^pro^ (75 nM) was preincubated in assay buffer containing 20 mM Tris, 150 mM NaCl, 1 mM EDTA, and 1 mM DTT, pH 7.3, for 10 min at 37 °C. Then, the enzyme was added to wells containing inhibitors (50 µg/mL essential oils or aromatic extracts), and the mixture was incubated for 30 min at 37 °C. After the incubation period, fluorogenic substrate (Ac-Abu-Tle-Leu-Gln-ACC) was added to the wells (final concentration 50 μM). ACC liberation was monitored for 30 min at 37 °C (λ_ex_ = 355 nm, λ_em_ = 460 nm) using a Molecular Devices Spectramax Gemini XPS spectrofluorometer. Each experiment was repeated twice (inhibition ≤ 50%) or five times (inhibition > 50%). The same experiments were performed for SARS-CoV-2 PL^pro^. SARS-CoV-2 PL^pro^ (150 nM) was preincubated in assay buffer containing 50 mM Tris, 5 mM NaCl, 0.075% BSA, and 5 mM DTT, pH 7.5, for 10 min at 37 °C. The assay conditions were the same as described above (Ac-Leu-Arg-Gly-Gly-ACC was used as the substrate to measure SARS-CoV-2 PL^pro^ residual activity).

### IC_50_ determination

Experimental conditions (concentration of enzyme and substrate, time) were tailored to obtain optimal enzyme activity during measurement like in our previous communications: PL^pro^^10^ and M^pro^^38^. Control experiments were carried out in the absence of the inhibitor (100% activity), enzyme or substrate. For selected inhibitors, the IC_50_ value was determined. Serial dilutions of inhibitors in assay buffer were prepared on 96-well plates (20 µL of each dilution in wells). SARS-CoV-2 M^pro^ (75 nM) or SARS-CoV-2 PL^pro^ (100 nM) was preincubated in assay buffer (20 mM Tris, 150 mM NaCl, 1 mM EDTA, 1 mM DTT, pH 7.3; 50 mM Tris, 5 mM NaCl, 0.075% BSA, 5 mM DTT, pH 7.5) for 10 min at 37 °C. Then, 60 µL of the enzyme was added to the wells containing serial dilutions of inhibitors (ranging from 1 µg/mL to 80 µg/mL), and the mixture was incubated for 30 min at 37 °C. After that time, 20 µL of the substrate (Ac-Abu-Tle-Leu-Gln-ACC for SARS-CoV-2 M^pro^ or Ac-Leu-Arg-Gly-Gly-ACC for SARS-CoV-2 PL^pro^) was added to each well. Measurements were carried out at 37 °C for 40 min (λ_ex_ = 355 nm, λ_em_ = 460 nm). The experiments were repeated three times. IC_50_ values were determined in GraphPad Prism software using nonlinear regression (dose–response – inhibition equation) and presented as relative enzyme activity vs. inhibitor concentration.

### Antiviral activity of essential oils and aromatic extracts toward a SARS-CoV-2 strain in VeroE6-GFP cell culture

VeroE6-GFP cells were seeded at a density of 25,000 cells/well in 96-well plates (Greiner Bio One, catalog no. 655090) and pretreated with threefold serial dilutions of the compounds overnight. On the next day (Day 0), cells were infected with the SARS-CoV-2 inoculum at a multiplicity of infection (MOI) of 0.001 tissue culture infectious dose (TCID_50_) per cell. The number of fluorescent pixels of the GFP signal determined by high-content imaging (HCI) on Day 4 postinfection (p. i.) was used as a read-out. The percent inhibition was calculated by subtracting the background (untreated-infected control wells) and normalizing to the untreated-uninfected control wells (also background subtracted). Experiments were carried out twice. The 50% effective concentration (EC_50_) was determined using logarithmic interpolation. The potential toxicity of compounds was assessed in a similar setup in treated-uninfected cultures, where metabolic activity was quantified at Day 5 using the MTS assay as described earlier^39^. The 50% cytotoxic concentration (CC_50_) was calculated by logarithmic interpolation.

### Gas chromatography (FID)

GC analyses were performed using a Shimadzu GC-2010 Plus gas chromatograph equipped with an FID detector and DB-5 (0.25 mm i.d. × 30 m, 0.25 μm film thickness, Agilent, Santa Clara, USA) capillary column. The injection port was maintained at 250 °C. The split ratio was set as 25:1, and 1 μL of the sample was injected. The oven temperature was set at 40 °C and increased to 300 °C at a rate of 2 °C/min, with a constant nitrogen carrier gas flow of 1.5 mL/min. The linear retention indices (RIs) of the compounds were calculated using the retention times of *n*-alkanes from C_8_ to C_26_.

### Gas chromatography–mass spectrometry (GC–MS)

GC–MS analyses were performed using an Agilent 7890A gas chromatograph equipped with an HP-5-MS capillary column (0.25 mm i.d. × 30 m, 0.25 μm film thickness, Agilent, Santa Clara, USA) combined with a WATERS GCT high-resolution mass spectrometer (TOF, EI +). The injection port was maintained at 250 °C. One microliter of the sample was injected in splitless mode. The oven temperature was held at 40 °C and raised to 300 °C at a rate of 2 °C/min, with a constant helium carrier gas flow of 1.5 mL/min. Mass spectra in electron impact (EI) mode were recorded at 70 eV ionization energy.

### Essential oil fractionation

Distillation of the petitgrain mandarin essential oil was carried out using a Buchi B-585 oven equipped with a Kugelrohr accessory. Eleven grams of the essential oil was placed in a 40 mL round-bottom flask and fractionated under a controlled distillation setup (2 mbar, 40–260 °C). Three fractions (3.22 g, 2.09 g, 3.91 g) and a residue (1.78 g) were analyzed using GC-FID and GC–MS.

## Results and discussion

### Natural flavorants and fragrances exhibit different inhibitory activities on both SARS-CoV-2 cysteine proteases.

A total of 538 samples of essential oils, aromatic extracts, and F&F raw materials of various origins were screened for their inhibitory properties on SARS-CoV-2 M^pro^ and PL^pro^. Screening (spectrofluorimetric enzymatic assay) was carried out with fluorogenic substrates designed by our group in previous works on SARS-CoV-2 proteases^10,37^. Detailed results for each material are presented in the supplementary data (Table S1). No or the lowest inhibitory activities were observed for all materials with high monoterpene and monoterpenoid contents. The most active natural flavorants and fragrances (inhibitory activity > 70%, Table 1) constitute mostly materials with both volatile and nonvolatile fractions, such as resinoids (storax, benzoin Sumatra and Siam, galbanum, and tolu), gums (elemi, storax, and labdanum), and absolutes (rocket, spinach, and oakmoss). The inhibitory potential of complex mixtures such as natural F&F materials is directly related to their composition, which depends on factors such as source plant species, country of origin, part of the plants used, and method of isolation. Clear differences can be observed, for example, between lovage root and leaf essential oils, where the former exhibits higher inhibitory activity on both SARS-CoV-2 proteases (77% and 39% for M^pro^ and PL^pro^, respectively, Table 1) than the latter (16% and 0% for M^pro^ and PL^pro^, respectively, Table S1, entry 52).

**Table 1:** Inhibitory activities of selected F&F materials against SARS-CoV-2 cysteine proteases.

English common name	Source plant botanical name	Main compounds of the natural product	Country of origin	M^pro^ (%)^a^	PL^pro^ (%)^a^
**Altingiaceae**					
Storax gum	*Liquidambar styraciflua*	Cinnamyl cinnamate 3-Phenylpropyl cinnamate	Honduras	90.5 ± 3.6	8
Storax resinoid	*Liquidambar orientalis*		Honduras	93.1 ± 0.7	13
**Amaranthaceae**					
Spinach absolute	*Spinacia oleracea*	Flavonoids	Egypt	76.6 ± 5.9	15
**Apiaceae**					
Galbanum resinoid	*Ferula gummosa Boiss* syn*. Ferula galbaniflua* Boiss. et Buhse	(+)-Nopinone (+)-Eremorphilene β-Amyrin	Iran	78.2 ± 0.8	11
Lovage root EO	*Levisticum officinale* Koch	*(Z)*-Ligustilide	Hungary	76.5 ± 1.9	39
**Brassicaceae**					
Rocket absolute	*Eruca vesicaria*	Erucic acid	Egypt	100	26
**Burseraceae**					
Elemi gum	*Canarium luzonicum* (Blume) A. Gray	β-Amyrin, brein, elemadienoic acid β-phellandrene Elemol Elemicin	Philippines	97.1 ± 1.9	17
**Cistaceae**					
Labdanum gum refined	*Cistus ladanifer*	Labdanoic acid	Spain	73.7 ± 2.2	0
**Cupressaceae**					
Blue cypress EO	*Callitris intratropica*	(+)-Bulnesol ( −)-Guaiol	Australia	73.1 ± 2.7	0
Cade crude *ex-sabine* EO	*Juniperus phoenicea*	α-Pinene δ − 3-Carene β-Phellandrene	Spain	94.0 ± 0.6	31
Siam wood EO	*Fokienia hodginsii*	Fokienol (*E*)-Nerolidol	Vietnam	83.8 ± 1.5	3
**Fabaceae**					
Cabreuva red EO	*Myrocarpus fastigiatus* Allemao	(*E*)-Nerolidol	Paraguay	78.6 ± 0.9	19
Tolu resinoid	*Myroxylon balsamum* (L.) Harms	Benzyl benzoate Benzyl cinnamate	Venezuela	77.3 ± 4.6	15
**Parmeliaceae**					
Oakmoss absolute	*Evernia Prunastri*	Evernin, Atranorin β-Orcinolcarboxylate	North Macedonia	71.4 ± 3.1	16
**Poaceae**					
Vetiver EO	*Chrysopogon zizanioides* (L.) Roberty syn. *Vetiveria zizanioides* (L.) Nash	Khusimol Vetiselinenol	Haiti	74.2 ± 4.3	0
**Rutaceae**					
Petitgrain mandarin (PM) EO	*Citrus reticulata* Blanco syn. *Citrus nobilis* Andrews	Dimethyl anthranilate	Egypt, Spain	85.3 ± 6.2	100
**Santalaceae**					
Sandalwood EO	*Santalum austro-caledonicum*	α-Santalol	New Caledonia	70.9 ± 3.3	4
**Styracaceae**					
Benzoin Siam resinoid	*Styrax tonkinensis*	Benzoic acid Benzoates	Laos	99.7 ± 0.4	17
Benzoin Sumatra resinoid	*Styrax benzoin*	Cinnamic acid Cinnamates	Indonesia	72.9 ± 2.5	9
**Zingiberaceae**					
Turmeric oleoresin	*Curcuma longa*	Curcuminoids β-Phellandrene *ar*-Turmerone	India	89.0 ± 0.7	64.3 ± 1.6
**Zygophyllaceae**					
Guaiacwood EO	*Bulnesia sarmientoi*	(+)-Bulnesol ( −)-Guaiol	Paraguay	72.0 ± 5.1	15

^a^Inhibitory activity of each F&F material at a concentration of 50 µg/mL. Numbers represent the mean value from two experiments (inhibition < 50%) or five experiments (inhibition > 50%).

Flavorants and fragrances derived from *Cistus ladanifer* are very good examples showing the distribution of activity within materials derived from one plant species. *Cistus ladanifer* is a pyrophoric plant and a source of highly prized labdanum gum. When the plant’s flowers begin to fade, the shrub develops leafy twigs. Its branches are covered with secretory hairs, which release abundant quantities of gum with an amber-like fragrance. The gum was historically harvested by combing goats, whose coats were covered with the exudate as they crisscrossed the hillsides^40^. Currently, cistus branches are gathered and processed industrially. Hydrodistillation or steam diffusion of cistus branches results in cistus traditional essential oil, which has a lemony, fresh, and amber-like olfactory profile. The addition of an extract from distillation water to the traditional oil gives ciste (full) essential oil with predominant amber-like, warm, and gourmand notes. Extraction of cistus twigs and branches with hydrocarbon-based solvent leads to a cistus concrete, which can be processed by ethanolic extraction that gives traditional cistus absolute with dry wood and sweet notes or by molecular distillation resulting in cistus SEV absolute with balsamic, smoky, and hot notes. Young cistus branches can also be dipped in a hot solution of sodium carbonate. This solution was acidified with sulfuric acid, and raw labdanum gum was obtained. Its hydrodistillation results in labdanum oil with intensive amber-like, woody notes. Extraction of gum with a hydrocarbon solvent leads to labdanum concrete, which after ethanolic extraction gives labdanum absolute, which possesses alcoholic, balsamic, and mild notes. Ethanolic extraction of raw gum leads to labdanum resinoids with balsamic, sweet, and amber-like olfactory profiles. The acid fraction of a mixture of labdanum gum and resinoid processed by molecular distillation leads to a labdasur-aroma material with animalic and cheese-like notes. It should be noted that despite having the same plant origin, cistus isolates present different olfactory properties. This means that some constituents are absent in one isolate but present in another, which may result in different biological activities. In this case, the inhibitory activities of various cistus isolates on SARS-CoV-2 M^pro^ are different, *e.g.,* labdanum gum—74%, labdanum resinoid—33%, ciste EO—34%, cistus absolute—0%, and cistus SEV absolute—42%.

In some materials, the nonvolatile fraction is mostly responsible for the inhibitory activity. This is evident in the case of elemi (*Canarium luzonicum*) gum (97% M^pro^ inhibition vs. 33% in the case of elemi essential oil) and galbanum (*Ferula galbaniflua*) resinoid (78% vs. 18% (EO) M^pro^ inhibition). Very illustrative examples of the influence of the method of isolation are turmeric-derived flavorants and fragrances, where the most likely polar volatile part is responsible for the M^pro^ inhibitory activity (essential oil—64%; isolation method: water/steam distillation vs. CO_2_ extract—33% inhibition) and the polar nonvolatile part for PL^pro^ inhibitory activity (essential oil—6% and CO_2_ extract—5% vs. oleoresin 64% inhibition). In the case of turmeric oleoresin, its polar nonvolatile fraction also enhances the M^pro^ inhibitory activity (89%).

*The Cuprassaceae* family constitutes a more abundant example of active essential oils. Cade crude *ex-sabine* EO was the most active (94%) against M^pro^, together with blue cypress and Siam wood essential oils. These activities were also retained for Chinese cedarwood (*Cupressus funebris*) and Virginian cedarwood (*Juniperus virginiana*) EOs (Table S1, 57% and 60% inhibition, respectively).

Most of the activity of the tested samples was attributed to the inhibition of the SARS-CoV-2 main protease. M^pro^ is the primary protease of the virus. The papain-like protease has a regulatory role, and both are essential for the processing of the polyprotein. In addition, PL^pro^ has an important role in counteracting the innate immunity of the host cell^9^. Ultimately, the best inhibitor would be the one that has high activities against one or both enzymes. There were only two examples (out of 400 tested) of such high activities—turmeric oleoresin (89% M^pro^ and 64% PL^pro^ inhibition) and petitgrain mandarin essential oil (85% M^pro^ and 100% PL^pro^ inhibition).

All the main compounds of oils, gums, and resinoids listed in Table 1 have been studied to determine whether they can be considered potent inhibitors of SARS-CoV-2 M^pro^ and SARS-CoV-2 PL^pro^. They were docked in the active center of the enzymes in the S1 cavity but with large binding freedom (20 Å) due to their large shape. Mostly they bind at the S1-S1′ cavity but are also shifted toward further cavities on the substrates and the products on both sides (Fig. S1A and B).

The structural similarity of the main compounds outside families is noted, and they can be divided into six main groups: benzoates and cinnamates, aliphatic acids, aromatic oxygenated compounds, low-molecular cyclic oxygenated compounds, terpene hydrocarbons, and terpenoid alcohols. The first group of compounds includes derivatives of organic acids based on the skeleton of benzoic or cinnamic acid. The extension of the ester or skeleton length could have a positive effect, increasing the inhibitory activity. Cinnamic acid esters could be more active than their shorter benzoate counterparts. Complex matrices of the remaining materials do not allow a direct comparison of the structure-inhibition relationship. The additional oxygen, hydroxy, or methoxy moieties or heteroatoms in other main groups of major constituents of the most active natural materials could not directly increase the inhibition of the enzyme. It is difficult to say whether it is certain, especially when a given natural product has an equal amount of individual ingredients.

However, the main constituents can be important for inhibitory activity; for example, (+)-bulnesol and (−)-guaiol prevail in blue cypress and guaiac wood essential oils. The results of inhibition for M^pro^ are almost the same, but for PL^pro^, the additional ingredients of guaiac wood oil increase its activity against this enzyme. The variation in the inhibition could be related to the different ratios of the two major compounds in blue cypress and guaiac wood essential oils.

### One compound contributes to the overall inhibitory activity of the petitgrain mandarin essential oil.

The essential oil isolated from mandarin leaves was the most potent in the preliminary screening studies. It comprises 6 main constituents (Table 2): α-pinene, β-pinene, *p*-cymene, (+)-limonene, γ-terpinene, and dimethyl anthranilate (DMA), with the last constituent being the most abundant.

**Table 2:** The main constituents of the petitgrain mandarin (*Citrus reticulata* blanco var. Mandarin) oils.

		RI^a^		Area^d^ [%]			Identification method^e^
		DB-5	Lit. DB-5	*EO*	*LFr*	*HFr*	
1	α-pinene	931	932^41^	2.19	3.65	2.42	RI, MS, ref
2	β-pinene	973	972^42^	2.18	3.51	1.92	RI, MS, ref
3	*p*-cymene	1023	1025^43^	14.7	9.09	2.19	RI, MS, ref
4	(+)-limonene	1027	1024^44^	8.26	11.3	8.34	RI, MS, ref
5	γ-terpinene	1057	1056^b^^45^	17.9	30.2	20.2	RI, MS, ref
6	dimethyl anthranilate (DMA)	1409	1407^c^^46^	43.5	35.1	60.6	RI, MS, ref

^a^Experimental and literature RIs relative to *n*-alkanes on a DB-5 capillary column unless otherwise stated. ^b^ RI for the DB-5MS column. ^c^ RI for the SLB-5MS column. ^d^ Area percentages of the main components; *EO* essential oil, *LFr* a light fraction of the oil, *HFr* a heavy fraction of the oil. ^e^Methods: *RI* identification based on RI comparison with literature data, *MS* identification based on mass spectra comparison with those of the Wiley Registry of Mass Spectral Data library (8th edition) and the Adams library of essential oil components (edition 4.1), *ref.* coinjection with an authentic sample.

Petitgrain mandarin essential oil was fractionated on Kugelrohr, three fractions and distillation residue were analyzed chromatographically, and their activities on both SARS-CoV-2 proteases were assessed (Table 3, the activity of fractions determined at the same concentration as the raw material during the screening phase). Before fractionation, the hypothesis was that the anthranilate part of the oil is responsible for the inhibitory activity (other materials with high monoterpene content exhibited low or no activity). The hypothesis was correct, as the inhibition rate increases with increasing dimethyl anthranilate content. The same can be observed for two fractions of PM essential oil (during the production of the oil, two fractions emerge—a fraction lighter than water, and a fraction heavier than water—the combination of both gives the commercial PM oil). This was further confirmed with the evaluation of pure natural dimethyl anthranilate, which fully inhibits both SARS-CoV-2 proteases under the same experimental conditions.

**Table 3:** Discovery of the most active constituents of the petitgrain mandarin (*Citrus reticulata* blanco var. Mandarin) oil against SARS-CoV-2 M^pro^ and PL^pro^.

		Area^a^ [%]					*LFr*	*HFr*	DMA
		*EO*	*Fr. 1*	*Fr. 2*	*Fr. 3*	*Res*			
1	α-pinene	2.19	4.07	0.7	0.03	0.0	3.65	2.42	–
2	β-pinene	2.18	4.36	1.2	0.03	0.0	3.51	1.92	–
3	*p*-cymene	14.7	27.5	20.2	0.6	0.06	9.09	2.19	–
4	limonene	8.26	16.4	9.9	0.2	0.03	11.3	8.34	–
5	γ-terpinene	17.9	33.5	27.1	0.8	0.05	30.2	20.2	–
6	dimethyl anthranilate (DMA)	43.5	7.0	33.8	93.0	92.7	35.1	60.6	100
M^pro^ inh. [%]		85	3	62	100	100	70	87	100
PL^pro^ inh. [%]		100	79	100	100	100	100	100	100

^a^Area percentages of the main components of the petitgrain mandarin oil and its fractions: *Fr.* distillation fraction number, *Res.* distillation residue, *EO* essential oil, *LFr* light fraction of the oil, *HFr* heavy fraction of the oil.

The detailed inhibition properties of the petitgrain mandarin essential oil, natural dimethyl anthranilate, and other most active essential oils and aromatic extracts were assessed (Table 4). As suspected from the fractionation studies, PM oil and DMA were significantly stronger inhibitors of SARS-CoV-2 papain-like protease. The best inhibitors of M^pro^ were benzoin Siam and Sumatra resinoids and rocket extracts (absolute and concrete) with IC_50_ values in range of 3.41 to 5.24 µg/mL.

**Table 4:** Inhibition parameters of essential oils and aromatic extracts and SARS-CoV-2 M^pro^ and PL^pro^.

Natural material	SARS-CoV-2 M^pro^ IC_50_ (µg/mL)	SARS-CoV-2 PL^pro^ IC_50_ (µg/mL) ([µM])
**Essential oils**		
Petitgrain mandarin EO	> 100	22.9 ± 4.6
DMA	> 100	5.20 ± 1.22 (31.5 ± 7.4)
Guaiacwood EO	48.01 ± 4.51	–
Blue cypress EO	48.22 ± 3.02	–
Lovage root EO	70.86 ± 4.65	–
Siam wood EO	76.10 ± 1.23	–
**Extracts**		
Benzoin Siam resinoid	5.24 ± 0.22	–
Benzoin Sumatra resinoid	4.20 ± 0.15	–
Galbanum resinoid	34.52 ± 2.27	–
Storax resinoid	46.31 ± 3.52	–
Turmeric oleoresin	12.01 ± 1.31	96.15 ± 2.09
Labdanum gum refined	47.44 ± 1.60	–
Nasturtium absolute	72.79 ± 1.12	–
Rocket absolute	3.41 ± 0.32	–
Rocket concrete	4.79 ± 0.04	–
*Tasmania lanceolata* extract	49.98 ± 1.99	–

Our exploratory study aimed to develop pioneering knowledge and provide the first experimental results on the inhibitory properties of hundreds of flavor and fragrance materials against SARS-CoV-2 main and papain-like proteases. The majority of available information is related to SARS-CoV. Only several plant extracts have been evaluated^47–49^ for their activity against the key proteases, and they exhibited moderate to low inhibitory potential (Table S2, entries 3–5) compared to two natural plant constituents, 3-isotheaflavin-3-gallate and tannic acid^50^, which had relatively good inhibitory activities against the SARS-CoV main protease (Table S2, entries 1 and 2).

### Molecular docking studies reveal the detailed binding method of DMA in the active sites of both enzymes.

**Figure 1:**
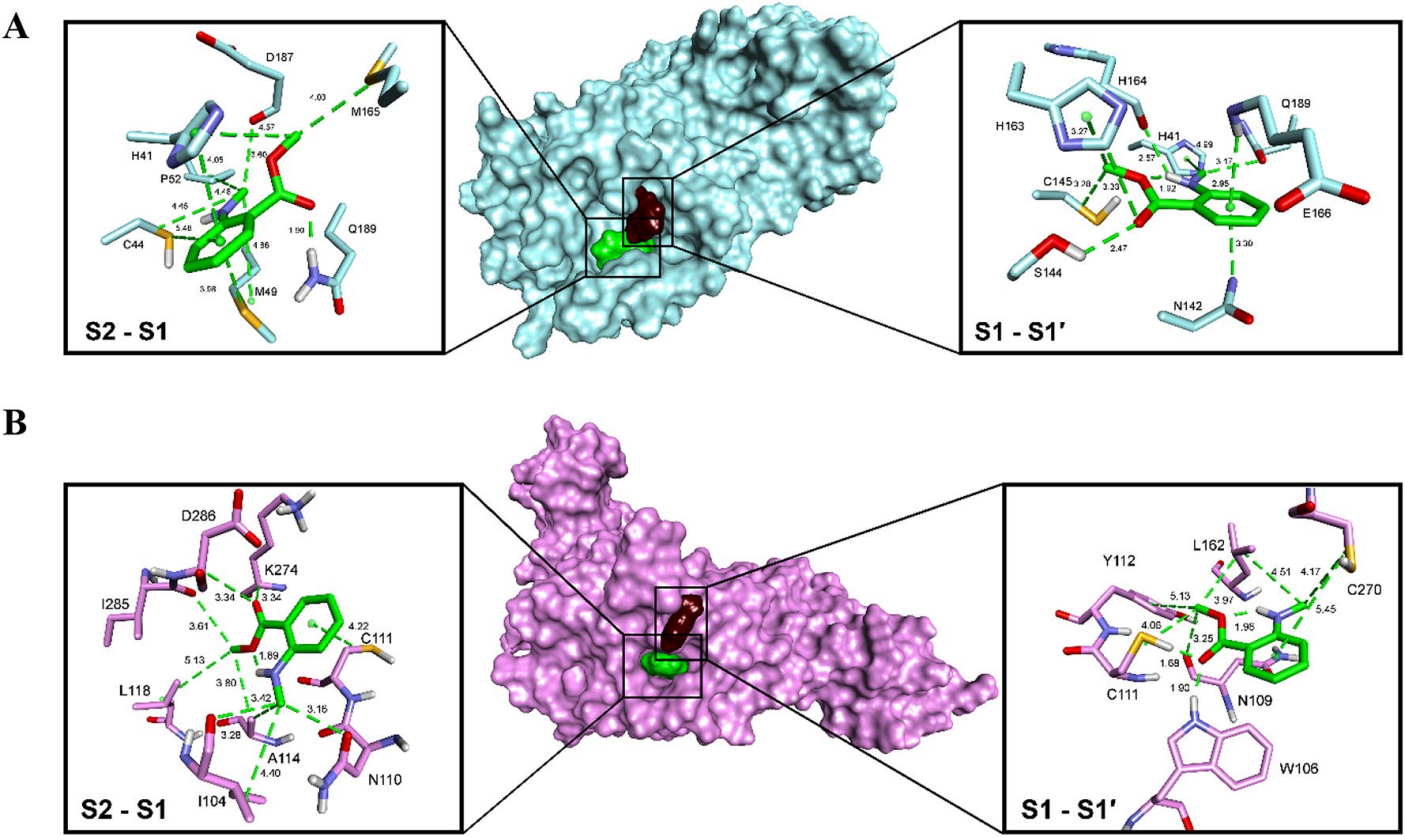
The interactions of dimethyl anthranilate in the active centers of SARS-CoV-2 M^pro^ (**A**) and SARS-CoV-2 PL^pro^ (**B**). The binding for each enzyme is shown for the S1-S1′ and S2-S1 pockets. The surface of the enzymes is colored light blue (M^pro^) or light pink (PL^pro^). The surface of methyl *N*-methylanthranilate is colored dark brown for the S1-S1′ pockets and green for the S2-S1 pockets. The ligands and the side chains of the amino acids are shown as sticks, and the bond order is not shown.

Dimethyl anthranilate was docked in the active centers of the enzymes to investigate ligand-enzyme interactions. Two types of binding were considered. The ligand was allowed to bind freely 20 Å from the catalytic cysteine (Cys145 for M^pro^ and Cys111 for PL^pro^) or was forced to interact with the mentioned amino acid. In the first step of docking, DMA interacted with the catalytic groups of SARS-CoV-2 M^pro^ in both docking pathways. The S1-S1′ pocket is not filled tightly by the ligand (Fig. 1A), which is directed with the methyl group of the ester to Cys145. The placement is stabilized by the hydrophobic interactions of Cys145–-Me (3.28 Å) and His163–-Me (3.27 Å). The carbonyl oxygen atom also interacts with histidine (3.33 Å). Its position is determined by a strong hydrogen bond with Ser144 (2.47 Å). Another strong hydrogen bond is noticeable for the amino group of the inhibitor with His164 (2.57 Å). The position of the phenyl ring and the *N*-methyl group seems to be rather constrained, and the interactions of the amino acids with them are weaker compared to the ester of the carboxyl group. In addition, their positions might be influenced by a strong intermolecular interaction of neighboring COOMe and NHMe groups (1.92 Å).

The optimization of the ligand-enzyme complex moved the dimethyl anthranilate toward the S2-S1 pocket (Fig. 1A). The phenyl ring finds itself in the middle of a hydrophobic area of a pocket built by His41 (4.05 Å), Cys44 (5.48 Å), and Met49 (3.98 Å). Intermolecular interactions are not formed because the side chain of Gln189 creates a strong hydrogen bond (1.90 Å) with the carbonyl oxygen atom, while the methyl ester group interacts with His41 (3.60 Å) and Met165 (4.03 Å). Such positioning of the ligand seems more natural and comparable to the enzyme–substrate complex. This is also confirmed by the large difference in the binding energy ΔG_S1-S1′_ = -16.70 kcal/mol vs. ΔG_S2-S1_ = -37.08 kcal/mol.

The amino acids building the S1 and S1′ active pockets of SARS-CoV-2 PL^pro^ are close, and the catalytic Cys111 is involved in the construction of both. Dimethyl anthranilate is placed very well in the S1 area (Fig. 1B). It creates strong hydrogen bonds between its carbonyl oxygen atom and the catalytic amino acids Trp106 (1.90 Å) and Cys111 (3.25 Å). The methyl ester group plays the role of a dovetail interlocking the compound in its position with hydrophobic interactions with Asn109 (3.25 Å), Tyr112 (5.13 Å), and Leu162 (3.97 Å). The leucine interacts hydrophobically with the *N*-methyl group (4.17 Å) likewise. A small role in the binding of the inhibitor is played by Cys270, which arranges the aromatic part of DMA (5.45 Å). Intramolecular interaction of COOMe and NHMe (1.96 Å) also occurs.

In the S2-S1 pocket, placement is different than in the M^pro^ (Fig. 1B). The phenyl ring interacts with Cys111 (4.22 Å), while Asn110 (3.16 Å) interacts with the hydrophobic amino acids Ile104 (4.40 Å, 3.28 Å for C=O–-Me) and Ala114 (3.42 Å). The alanine (3.80 Å), Leu118 (5.13 Å) and Ile285 (3.61 Å) position the methyl carboxylate. The carbonyl oxygen atom interacts with the carbonyl oxygen atom of Asp286 (3.34 Å) and through the carbon–hydrogen bond with Lys274 (3.34 Å), but those bonds come from recurring intermolecular interactions (1.89 Å). The binding energy determines that the S2-S1 pocket is also preferred: ΔG_S1-S1′_ = − 17.02 kcal/mol vs. ΔG_S2-S1_ = − 23.59 kcal/mol. The compound binds well in both selected areas of both tested enzymes; however, the binding energy confirms that they prefer S2-S1.

**Figure 2:**
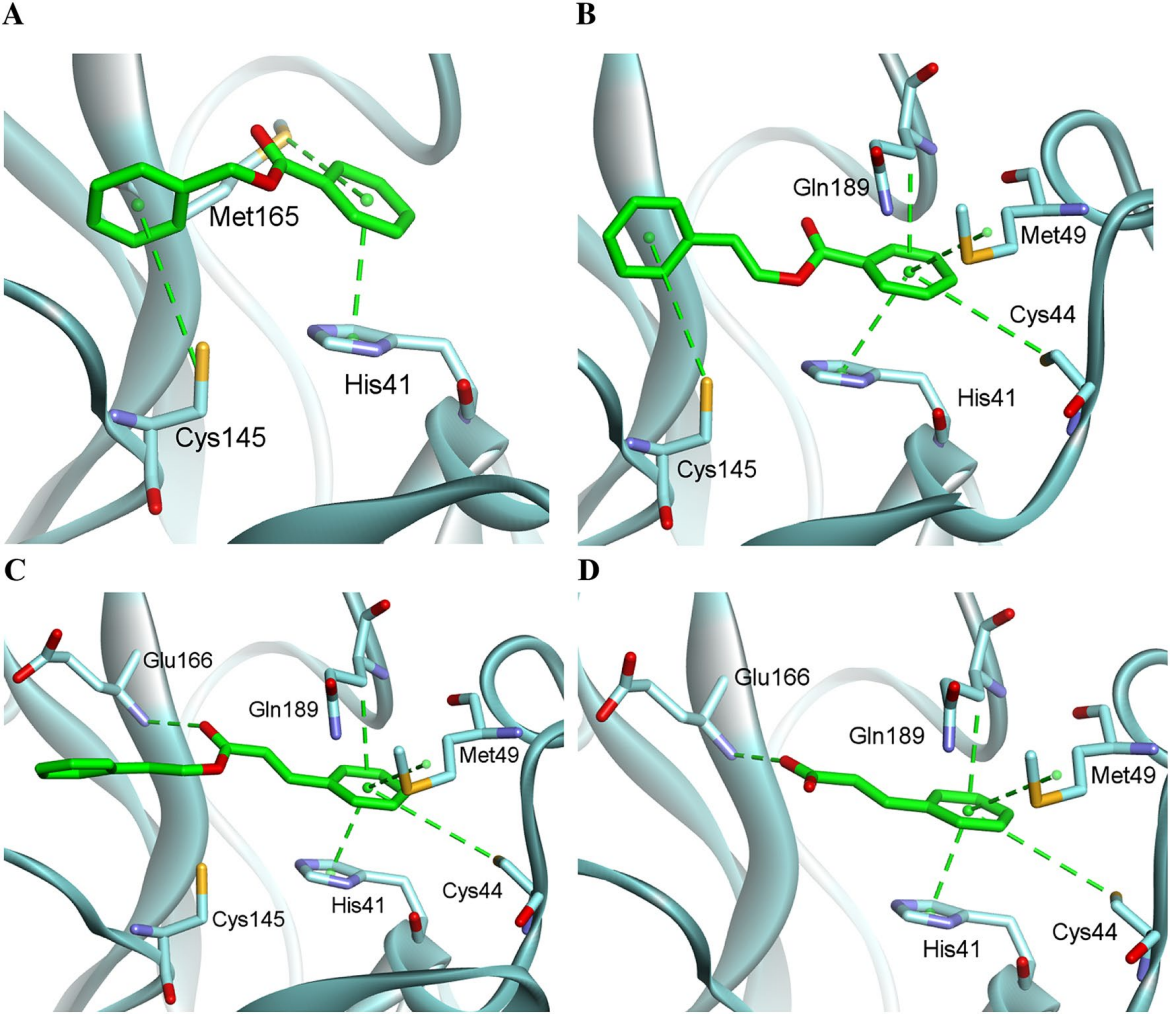
The interactions of benzyl benzoate (**A**), benzyl cinnamate (**B**), cinnamyl cinnamate (**C**), and cinnamic acid (**D**) in the active center of SARS-CoV-2 M^pro^. The ligands and the side chains of the amino acids are shown as sticks, and the bond order is not shown.

Benzoin resinoids (Siam and Sumatra) demonstrated very promising inhibitory activity against M^pro^. They are comprised of derivatives of benzoic and cinnamic acids. Four main constituents were evaluated in silico on SARS-CoV-2 M^pro^ (Fig. 2): benzyl benzoate (Fig. 2A), benzyl cinnamate (Fig. 2B), cinnamyl cinnamate (Fig. 2C), and cinnamic acid (Fig. 2D). For the general nature of the inhibition, we assumed that the unsaturated parts of the compounds are *trans*-isomers.

Benzyl derivatives (Fig. 2A and B) are directed by the phenyl ring of the acid part toward the deep part of the S2 pocket of the active center. Arenes are stabilized by multiple hydrophobic interactions with lipophilic amino acids, such as His41 and Met49. However, none of the amino acids create hydrogen bonds between the ligand and protein. Hydrogen interaction occurs when the backbone is elongated. Carbonyl oxygen interacts with the amino group’s hydrogen atom from Glu166. The attracted ligands are too distant to form a weak phenyl-Cys145 interaction. Nevertheless, it may expose the double bond from the ester and create the possibility of forming a covalent bond between the aforementioned cysteine and the backbone of the compound.

### Benzoin Sumatra resinoid has the most prominent antiviral activity.

The most active protease inhibitors were also tested for their antiviral activity using a fully replication-competent SARS-CoV-2 strain in VeroE6-GFP cell culture (Table S3). The benzoin Sumatra resinoid showed selective inhibition (half-maximal effective concentration (EC_50_) = 31.5 ± 2.4 µg/mL) with limited toxicity (50% cytotoxic concentration (CC_50_) = 85.5 ± 1.9 µg/mL). The best PL^pro^ inhibitor, petitgrain mandarin essential oil, showed lower antiviral activity (> 100 µg/mL).

Available data on the activity of plant materials against highly pathogenic human coronaviruses are scarce. A few studies have been conducted on the anti-SARS-CoV activities of plant extracts and essential oils (Table S4). Evaluation of cinnamon bark and clove bud aqueous extracts (Table S4, entries 1–5) showed that the hydroalcoholic extract of cinnamon bark and its butanol fraction possess moderate inhibitory activity against SARS-CoV infection^51^. Screening of 121 Chinese herbs resulted in the identification of two compounds (Table S4, entries 6 and 7), tetra-*O*-galloyl-β-D-glucose from *Galla chinensis* and luteolin from *Veronica linariifolia*, which could inhibit SARS-CoV infection in a dose-dependent manner^52^. Only one reference addresses the evaluation of anti-SARS-CoV activities of essential oils^53^ (Table S4, entries 10–16), with laurel EO being the most active (modestly).

In addition to the in vitro evaluations, several randomized controlled trials showed some promise of Chinese herbal medicine combined with conventional medicine for the treatment of SARS, but the evidence was insufficient due to the low methodological quality of the trials^54^.

Relevant data on the activity of plant materials against MERS-CoV and SARS-CoV-2 are even more scarce, as most of the references are focused on in silico evaluations of natural products (including essential oil constituents)^55^ or perspectives in this field^56^. Two of the few meaningful references describe the antiviral value of the hydroalcoholic extract of *Echinacea purpurea* herb and roots (Echinaforce^®^) against MERS-CoV and SARS-CoV-2^57^. Fifty micrograms/ml Echinaforce^®^ (the final concentration of ethanol was 0.2%, and it was proven that the tested coronaviruses were not sensitive to this residual concentration) fully blocked the infectivity of MERS-CoV and SARS-CoV-2. Most recently, one study described the anti-SARS-CoV-2 activity of some *Lamiaceae* EOs and their monoterpene constituents^58^. The most active essential oil (*Mentha vilosa*) exhibited moderate inhibitory activity (127.00 ± 4.63 ppm), as did carvacrol (80.23 ± 6.07 µM). The main omission of the abovementioned study is the lack of stereochemical descriptors of the evaluated optically active monoterpenes. In addition, toxicological aspects of pennyroyal (*Mentha pulegium*) essential oil, menthofuran, and pulegone are not discussed in enough detail. The results clearly show no or limited toxicity of the abovementioned materials to Vero 76 cells, but they are known to be highly toxic to humans.

Despite the lack of experimental in vitro data on the potential of flavor and fragrance materials against SARS-CoV-2, two clinical trials evaluated the potential of a commercial product containing essential oil constituents (Listerine^®^—originally EO-based, now comprising a mixture of eucalyptol, (−)-menthol, thymol, and methyl salicylate) to eliminate SARS-CoV-2 in the throat and nasopharynx of COVID-19 patients. The first trial (^59^, NCT04410159) was completed and showed high viral clearance [two negative RT–PCR (of swabs from the oropharynx and nasopharynx) results at least 24 h apart] rates for 1% iodine-povidone (Betadine^®^) and “essential oils” (Listerine^®^) gargles (100% and 80%, respectively) 4 days after intervention (median time without intervention—14 days). These results should be treated with caution, as the testing groups were very small (5 patients in each gargle group). The second “gargle” clinical trial (NCT04584684), “Antiviral Efficacy and Acceptability of Therapeutic Antiseptic Mouth Rinses for Inactivation of COVID SARS-2 Virus”, is ongoing. Its goal is to test the efficacy of antiseptic mouth rinses (*e.g.,* Listerine^®^) to inactivate SARS-CoV-2 in the saliva of COVID-19-positive patients (480 patients). Listerine^®^ is a hydroalcoholic mixture of essential oil constituents. The ethanol concentration is at the level of 21%, which is significant, but it has been proven that the concentration of ethyl alcohol for the complete inactivation of SARS-CoV-2 must be ≥ 30% (^60^, 20% EtOH concentration resulted in a reduction factor of only 1.1).

## Conclusion

F&F materials are important for society because they are commonly used in various areas of human interest, have established toxicological profiles and are relatively safe. Until recently, the tools to assess the inhibitory potential of chemicals against the key SARS-CoV-2 proteases were limited. Our recent developments^10,37^ provided a framework for the discovery of new materials/molecules with potential therapeutic value. The results of this study give the scientific community the selection criteria for further in vitro and in vivo testing of the most promising EOs, AEs, and isolated natural compounds. They can also be used for the rational design of SARS-CoV-2 protease inhibitors. Even though important countermeasures for the spread of SARS-CoV-2 are in motion (i.e., the introduction of vaccines), these results will be important to the field because though coronaviruses can change, the coronaviral proteases are conserved (broad application of results in potential future outbreaks).

## Supplementary Information

Supplementary Information.

## Data Availability

The data that support the findings of this study are available from the corresponding author upon reasonable request.

## Abbreviations

Abu: 2-Aminobutanoic acid
ACC: 7-Amino-4-carbamoylmethylcoumarin
RFU: Relative fluorescence unit
Tle: *Tert*-Leucine
EO: Essential oil
AE: Aromatic extract
MBTK: Manuka oil beta triketones
DMA: Dimethyl anthranilate
