# Supplementary material for: Evaluation of the anti-SARS-CoV-2 properties of essential oils and aromatic extracts
Source: Sci Rep. 2022 Aug 20;12:14230. doi: 10.1038/s41598-022-18676-w (PMC9392441; doi:10.1038/s41598-022-18676-w)
Supplement: Supplementary file 1 — Supplementary Information. [file 41598_2022_18676_MOESM1_ESM.docx]

**Evaluation of the anti-SARS-CoV-2 properties of essential oils and aromatic extracts**

Daniel Jan Strub^1,2,*^, Michał Talma^3^, Maria Strub^2^, Wioletta Rut^1^, Mikolaj Zmudzinski^1^, Władysław Brud^4^, Dirk Jochmans^5^, Laura Vangeel^5^, Linlin Zhang^6^, Xinyuanyuan Sun^6^, Zongyang Lv^7,8^, Digant Nayak^7,8^, Shaun K. Olsen^7,8^, Rolf Hilgenfeld^6,9^, Johan Neyts^5^, Marcin Drąg^1*^

*^1^Department of Chemical Biology and Bioimaging, Wrocław University of Science and Technology, Wyb. Wyspiańskiego 27, 50-370 Wrocław, Poland*

*^2^ Liquid Technologies sp. z o.o., Gdańska 13, 50-344 Wrocław, Poland*

*^3^ Department of Bioorganic Chemistry, Wrocław University of Science and Technology, Wyb. Wyspiańskiego 27, 50-370 Wrocław, Poland*

*^4^ Scientific Committee of the International Federation of Essential Oils and Aroma Trades (IFEAT), c/o TC Group, Level 1, Devonshire House, One Mayfair Place, London W1J 8AJ, UK*

*^5^ Laboratory of Virology and Chemotherapy, Department of Microbiology, Immunology and Transplantation, Rega Institute, KU Leuven, Leuven, Belgium*

*^6^ Institute of Molecular Medicine, University of Lübeck, Ratzeburger Allee 160, 23562 Lübeck, Germany*

*^7^ Department of Biochemistry and Molecular Biology and Hollings Cancer Center, Medical University of South Carolina, Charleston, SC 29425, USA.*

*^8^ Department of Biochemistry and Structural Biology University of Texas Health Science Center at San Antonio, San Antonio, TX 78229, USA.*

*^9^ German Center for Infection Research (DZIF), Hamburg-Lübeck-Borstel-Riems Site, University of Lübeck, 23562 Lübeck, Germany*

corresponding authors: daniel.strub@pwr.edu.pl, marcin.drag@pwr.edu.pl

[**Modeling of the main compounds of the natural products onto crystal structures of SARS-CoV-2 proteases** 2](#_Toc92109168)

[**Inhibition assay of selected F&F materials** 3](#_Toc92109169)

[**Inhibitory activity of plant extracts against SARS-CoV-1 proteases** 31](#_Toc92109170)

[**Antiviral assay of selected F&F materials** 32](#_Toc92109171)

[**Anti-SARS-CoV-1 activity of essential oils and plant extracts** 33](#_Toc92109172)

[**IC_50_ graphs for PL^pro^** 34](#_Toc92109173)

[**IC_50_ graphs for M^pro^** 35](#_Toc92109174)

[**References** 37](#_Toc92109175)

# **Modeling of the main compounds of the natural products onto crystal structures of SARS-CoV-2 proteases**

| **A** | **B** |
| --- | --- |
| **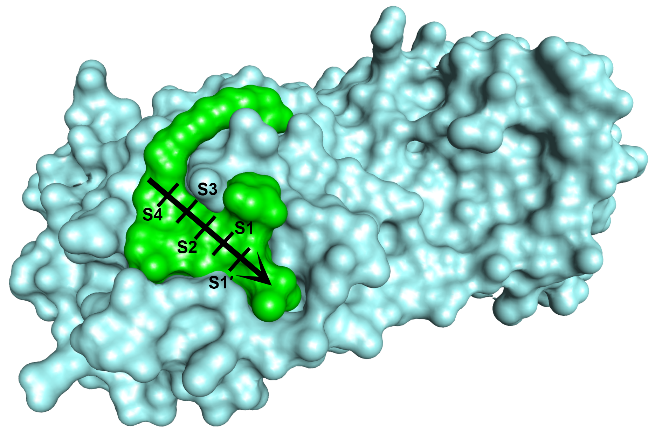** | **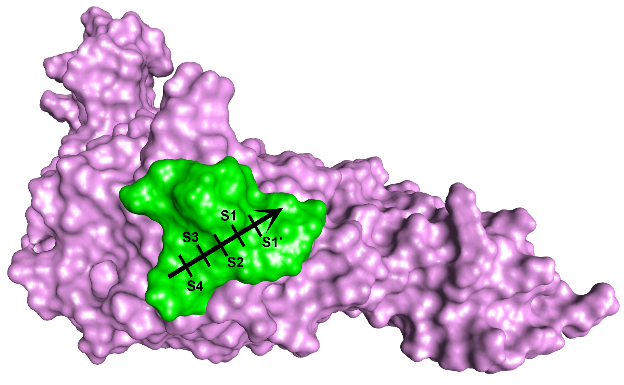** |
|  | |

Figure S1. Modeling of the main compounds of the natural products onto crystal structures of SARS-CoV-2-M^pro^ (A, PDB: 6XBH) and SARS-CoV-2-PL^pro^ (B, PDB: 6WX4) with approximate scaling of the S4-S1′ active cavities. Ligands surface is colored green and represents the total volume of all of the studied compounds in the active centers. The surface of the enzymes is colored light blue (M^pro^ ) or light pink (PL^pro^).

# **Inhibition assay of selected F&F materials**

Table S1. Inhibition activity of selected F&F materials against SARS-CoV-2 cysteine proteases. ^*^ Inhibitory activity for the concentration of 50 µg/mL of each F&F material. Numbers represent the mean value from two experiments (inhibition < 50%) or five experiments (inhibition > 50%).

| **No.** | **English common name** | **Botanical name** | **Country of origin** | **M^pro *^**  **[%] inh.** | **PL^pro^ ***  **[%] inh.** |
| --- | --- | --- | --- | --- | --- |
| *Achariaceae* | | | | | |
| 1 | Chaulmoogra seed oil (exp.) | *Hydnocarpus wightianus* | India | 0 | 22 |
| *Acoraceae* | | | | | |
| 2 | Sweet flag  Calamus EO | *Acorus calamus* | India | 1 | 0 |
| *Altingiaceae* | | | | | |
| 3 | Storax EO | *Liquidambar styraciflua* | Honduras | 17 | 15 |
| 4 | Storax gum |  | Honduras | 90.5±3.6 | 8 |
| 5 | Storax gum |  | Guatemala | 68.8±5.9 | 9 |
| 6 | Storax gum raw |  | Guatemala | 68.7±4.4 | 15 |
| 7 | Storax resinoid |  | Honduras | 93.1±0.7 | 13 |
| 8 | Storax resinoid | *Liquidambar orientalis* | Turkey | 49 | 2 |
| *Amaranthaceae* | | | | | |
| 9 | Spinach absolute | *Spinacia oleracea* | Egypt | 76.6±5.9 | 15 |
| *Amaryllidaceae* | | | | | |
| 10 | Garlic EO | *Allium sativum* | China | 47 | 34 |
| 11 | Leek EO | *Allium porum* | Egypt | 46 | 23 |
| 12 | Onion EO | *Allium cepa* | Egypt | 40 | 15 |
| *Anacardiaceae* | | | | | |
| 13 | Mango leaf absolute | *Mangifera indica* | Egypt | 22 | 14 |
| 14 | Mastic EO | *Pistacia lentiscus* | Greece | 22 | 0 |
| 15 | Mastic EO |  | Morocco | 18 | 19 |
| 16 | Pepper pink CO_2_ | *Schinus terebinthifolia* Raddi | Madagascar | 13 | 6 |
| 17 | Schinus molle EO | *Schinus molle* | Peru | 0 | 5 |
| *Annonaceae* | | | | | |
| 18 | Cananga EO | *Cananga odorata (Lam.)*  Hook. f. & Thomson  forma *macrophylla* | Indonesia | 39 | 6 |
| 19 | Ylang-Ylang complete EO | *Cananga odorata* Hook. f. & Thomson forma *genuina* | Madagascar | 0 | 6 |
| 20 | Ylang-Ylang Extra EO |  | Madagascar | 28 | 22 |
| 21 | Ylang-Ylang I EO |  | Comoros | 0 | 23 |
| 22 | Ylang-Ylang I EO |  | Madagascar | 5 | 23 |
| 23 | Ylang-Ylang II EO |  | Comoros | 6 | 20 |
| 24 | Ylang-Ylang II EO |  | Madagascar | 24 | 4 |
| 25 | Ylang-Ylang III EO |  | Comoros | 7 | 13 |
| 26 | Ylang-Ylang III EO |  | Madagascar | 47 | 25 |
| *Apiaceae* | | | | | |
| 27 | Ajowan EO | *Trachyspermum ammi*  (L.) Sprague ex Turill | India | 0 | 0 |
| 28 | Angelica root EO | *Angelica archangelica* | Hungary | 1 | 4 |
| 29 | Angelica root EO |  | India | 56.6±5.7 | 76.9±3.4 |
| 30 | Angelica seed EO |  | USA | 8 | 3 |
| 31 | Anise seed EO | *Pimpinella anisum* | Egypt | 12 | 5 |
| 32 | Anise seed EO |  | Greece | 4 | 2 |
| 33 | Asafoetida EO | *Ferula assa-foetida* | India | 0 | 14 |
| 34 | Caraway seed EO | *Carum carvi* | Hungary | 0 | 1 |
| 35 |  |  | Egypt | 0 | 14 |
| 36 | Caraway seed absolute |  |  | 0 | 25 |
| 37 | Carrot leaf absolute | *Daucus carota* | Egypt | 0 | 10 |
| 38 | Carrot seed EO |  | Greece | 14 | 0 |
| 39 | Carrot seed EO |  | France | 38 | 0 |
| 40 | Carrot seed EO |  | India | 29 | 12 |
| 41 | Carrot seed EO |  | Egypt | 35 | 0 |
| 42 | Carrot seed absolute |  |  | 28 | 26 |
| 43 | Celery herb EO | *Apium graveolens* | Egypt | 0 | 0 |
| 44 | Celery herb EO |  | France | 18 | 0 |
| 45 | Celery herb EO |  | India | 0 | 6 |
| 46 | Celery herb EO |  | Egypt | 0 | 0 |
| 47 | Coriander leaves EO | *Coriandrum sativum* | Russia | 0 | 14 |
| 48 | Coriander leaves EO |  | Egypt | 1 | 0 |
| 49 | Coriander leaves absolute |  |  | 33 | 24 |
| 50 | Coriander fruits EO |  | Egypt | 0 | 0 |
| 51 | Coriander fruits EO |  | Greece | 13 | 0 |
| 52 | Coriander fruits EO |  | Russia | 2 | 11 |
| 53 | Cumin EO | *Cuminum cyminum* | Egypt | 0 | 20 |
| 54 | Cumin absolute |  |  | 1 | 21 |
| 55 | Cumin EO |  |  | 20 | 21 |
| 56 | Cumin oleoresin |  | Spain | 0 | 12 |
| 57 | Dill seed EO | *Anethum graveolens* | South Africa | 2 | 0 |
| 58 | Dill seed EO |  | Greece | 22 | 5 |
| 59 | Dill weed EO |  | Austria | 0 | 5 |
| 60 | Dill weed EO |  | Egypt | 0 | 5 |
| 61 | Dill weed EO |  | USA | 16 | 11 |
| 62 | Fennel bitter EO | *Foeniculum vulgare*  Mill. subsp. *vulgare* var.  *vulgare* | Australia | 18 | 0 |
| 63 | Fennel bitter 70% fenchone EO |  | Australia | 27 | 7 |
| 64 | Fennel bitter EO |  | Spain | 0 | 0 |
| 65 | Fennel bitter EO |  | Egypt | 0 | 18 |
| 66 | Fennel sweet EO | *Foeniculum vulgare* Mill.  subsp. *vulgare* var. *dulce*  (Mill.) Batt | Australia | 24 | 24 |
| 67 | Fennel sweet EO |  | Greece | 17 | 0 |
| 68 | Fennel sweet EO |  | Hungary | 14 | 10 |
| 69 | Fennel sweet EO |  | Egypt | 0 | 15 |
| 70 | Galbanum EO | *Ferula gummosa* Boiss.  syn. *Ferula galbaniflua*  Boiss. et Buhse | Iran | 11 | 9 |
| 71 | Galbanum resinoid | *Ferula gummosa* Boiss.  syn. *Ferula galbaniflua*  Boiss. et Buhse | Iran | 78.2±0.8 | 11 |
| 72 | Lovage leaf EO | *Levisticum officinale* Koch | Hungary | 16 | 0 |
| 73 | Lovage root EO |  | Hungary | 76.5±1.9 | 39 |
| 74 | Parsley herb EO | *Petroselinum crispum*  (Mill.) Fuss  syn. *Petroselinum sativum* Hoffm | Australia | 42 | 0 |
| 75 | Parsley herb EO |  | Egypt | 49 | 18 |
| 76 | Parsley fruit EO |  | Australia | 26 | 28 |
| 77 | Parsley fruit EO |  | Egypt | 11 | 5 |
| 78 | Parsley fruit EO |  | Romania | 0 | 15 |
| 79 | Sea fennel EO | *Crithmum maritimum* | Greece | 49 | 0 |
| *Aquifoliaceae* | | | | | |
| 80 | Mate absolute | *Ilex Paraguariensis* | Brazil | 0 | 5 |
| *Asparagaceae* | | | | | |
| 81 | Tuberose absolute | *Polianthes tuberosa* | India | 0 | 18 |
| *Asteraceae* | | | | | |
| 82 | Armoise EO | *Artemisia herba-alba* Asso. | North Africa | 0 | 14 |
| 83 | Artichoke absolute | *Cynara cardunculus* | Egypt | 39 | 41 |
| 84 | Blue tansy EO | *Tanacetum annuum* | Morocco | 7 | 0 |
| 85 | Calendula absolute | *Calendula officinalis* | Egypt | 21 | 41 |
| 86 | Calendula concrete |  |  | 49 | 28 |
| 87 | Chamomile blue EO | *Matricaria chamomilla* L.  syn. *Chamomilla recutita*  (L.) Rauschert | Egypt | 2 | 0 |
| 88 | Chamomile blue EO |  | Hungary | 18 | 0 |
| 89 | Chamomile blue EO |  | Greece | 31 | 0 |
| 90 | Chamomile blue absolute |  | Egypt | 0 | 31 |
| 91 | Chamomile blue concrete |  |  | 4 | 31 |
| 92 | Chamomile Roman EO | *Chamaemelum nobile*  (L.) All.  syn. *Anthemis nobilis* L.  syn. *Ormenis nobilis* (L.)  J. Gay ex Coss. & Germ. | France | 0 | 0 |
| 93 | Chamomile Roman EO |  | Greece | 9 | 0 |
| 94 | Chamomile wild EO | *Cladanthus mixtus* (L.)  Chevall.  syn. *Chamaemelum mixtum* (L.) All. | Morocco | 8 | 2 |
| 95 | Cotula EO | *Cotula cinerea* | Egypt | 21 | 29 |
| 96 | Davana EO | *Artemisia pallens* Wall.  ex DC. | India | 25 | 15 |
| 97 | Everlasting EO | *Helichrysum italicum*  (Roth) G. Don  syn. *Helichrysum angustifolium* (Lam.) DC. | Spain | 24 | 9 |
| 98 | Everlasting EO |  | Greece | 18 | 2 |
| 99 | Goldenrod EO | *Solidago canadensis* | Canada | 4 | 8 |
| 100 | Helichrysum EO | *Helichrysum gymnocephalum* | Madagascar | 14 | 23 |
| 101 | Helichrysum EO | *Helichrysum bracteiferum* | Madagascar | 31 | 20 |
| 102 | Iary EO | *Psiadia altissima* | Madagascar | 31 | 0 |
| 103 | Inula EO | *Inula graveolens* | France | 20 | 0 |
| 104 | Irish lace (anis del monte) EO | *Tagetes filifolia* | Ecuador | 26 | 50 |
| 105 | Milfoil (yarrow) EO, 3% chamazulene | *Achillea millefolium* | Austria | 43 | 17 |
| 106 | Milfoil (yarrow) EO, 15% chamazulene |  | Austria | 40 | 16 |
| 107 | Milfoil (yarrow) EO |  | Bulgaria | 41 | 27 |
| 108 | Milfoil (yarrow) EO |  | Greece | 11 | 3 |
| 109 | Milfoil (yarrow) EO |  | Spain | 28 | 0 |
| 110 | Tagete EO | *Tagetes minuta* L.  syn. *Tagetes glandulifera* Schrank | Egypt | 48 | 28 |
| 111 | Tagete absolute |  |  | 39 | 24 |
| 112 | Tagete EO |  | Madagascar | 16 | 19 |
| 113 | Tagete EO |  | Mexico | 14 | 0 |
| 114 | Tagete EO |  | South Africa | 1 | 0 |
| 115 | Tagete EO |  | Zimbabwe | 59.1±1.7 | 0 |
| 116 | Tarragon EO | *Artemisia dracunculus* | USA | 0 | 15 |
| 117 | Tarragon EO |  | Spain | 0 | 8 |
| 118 | Wormwood herb EO | *Artemisia absinthium* | Russia | 0 | 0 |
| 119 | Wormwood herb EO |  | USA | 0 | 16 |
| *Betulaceae* | | | | | |
| 120 | Birch tar EO | *Betula pendula* Roth | France | 45 | 7 |
| *Brassicaceae* | | | | | |
| 121 | Rocket absolute | *Eruca vesicaria* | Egypt | 100±0.1 | 26 |
| 122 | Rocket concrete |  |  | 94 | 33 |
| *Burseraceae* | | | | | |
| 123 | Copal EO | *Protium amazonicum* | Ecuador | 18 | 21 |
| 124 | Elemi EO | *Canarium luzonicum*  (Blume) A. Gray | Phillipines | 33 | 31 |
| 125 | Elemi gum | *Canarium luzonicum*  (Blume) A. Gray | Phillipines | 97.1±1.9 | 17 |
| 126 | Myrrh EO | *Commiphora myrrha*  (Nees) Engl.  syn. *C. molmol* (Engl.)  Engl. ex Tschirch | Somalia | 68.2±0.9 | 19 |
| 127 | Myrrh resinoid |  |  | 2 | 15 |
| 128 | Myrrh resin |  |  | 23 | 31 |
| 129 | Olibanum (frankincense) EO | *Boswellia sacra* Flueck.  syn. *Boswellia carteri*  Birdw. | Somalia | 21 | 20 |
| 130 | Olibanum (emerald frankincense) EO |  | Somalia | 27 | 0 |
| 131 | Olibanum (frankincense) EO |  | Ethiopia | 31 | 0 |
| 132 | Olibanum (frankincense) EO |  | India | 10 | 0 |
| 133 | Olibanum (frankincense) EO |  | Oman | 26 | 0 |
| 134 | Olibanum (frankincense) resinoid |  | Somalia | 14 | 5 |
| 135 | Opopanax EO | *Commiphora erythraea*  (Ehrenb.) Engl. | Somalia | 0 | 11 |
| 136 | Opopanax absolute |  |  | 0 | 9 |
| 137 | Opopanax resinoid |  |  | 0 | 15 |
| 138 | Palo Santo EO | *Bursera graveolens* | Ecuador | 27 | 34 |
| *Cannabaceae* | | | | | |
| 139 | Hops EO | *Humulus lupulus* | Austria | 26 | 11 |
| 140 |  |  | Canada | 14 | 15 |
| *Caprifoliaceae* | | | | | |
| 141 | Honeysuckle absolute | *Lonicera caprifolium* | Egypt | 0 | 14 |
| 142 | Spikenard EO | *Nardostachys grandiflora* DC.  syn. *Nardostachys jatamansi* (D.Don) DC. | India | 30 | 5 |
| *Caryophyllales* | | | | | |
| 143 | Carnation absolute | *Dianthus caryophyllus* | Egypt | 0 | 8 |
| 144 | Carnation concrete |  |  | 3 | 12 |
| *Cistaceae* | | | | | |
| 145 | Cistus complete EO | *Cistus ladanifer* | Spain | 34 | 22 |
| 146 | Cistus EO |  | Spain | 24 | 13 |
| 147 | Cistus EO |  | Greece | 3 | 2 |
| 148 | Cistus absolute |  | Spain | 0 | 20 |
| 149 | Cistus SEV absolute |  | Spain | 42 | 11 |
| 150 | Labdacistus EO |  | Spain | 0 | 0 |
| 151 | Labdanum absolute |  | Spain | 48 | 20 |
| 152 | Labdanum gum refined |  | Spain | 73.7±2.2 | 0 |
| 153 | Labdanum resinoid |  | Spain | 33 | 12 |
| *Cupressaceae* | | | | | |
| 154 | Amber solid | *Juniperus Virginiana* | USA | 59.4±2.9 | 53.9±1.9 |
| 155 | Blue cypress EO | *Callitris intratropica* | Australia | 73.1±2.7 | 0 |
| 156 | Cade crude *ex-sabine* EO | *Juniperus phoenicea* | Spain | 94.0±0.6 | 31 |
| 157 | Cade, rectified EO | *Juniperus oxycedrus* | India | 34 | 4 |
| 158 | Cedarleaf EO | *Thuja occidentalis* | Canada | 0 | 10 |
| 159 | Cedarwood EO | *Cupressus funebris* | China | 56.7±4.5 | 0 |
| 160 | Cedarwood Texas EO | *Juniperus ashei* J. Buchholz | USA | 38 | 18 |
| 161 | Cedarwood Virginian EO | *Juniperus virginiana* | USA | 60.4±2.9 | 0 |
| 162 | Cypress EO | *Cupressus sempervirens* | Italy | 17 | 14 |
| 163 | Cypress EO |  | Greece | 27 | 2 |
| 164 | Cypress absolute |  | Italy | 39 | 12 |
| 165 | Hinoki EO | *Chamaecyparis obtusa* | Japan | 0 | 18 |
| 166 | Juniper berry EO | *Juniperus communis* | North Macedonia | 0 | 6 |
| 167 | Juniper berry EO |  | Greece | 14 | 7 |
| 168 | Juniper Berry CO_2_ |  | India | 35 | 0 |
| 169 | Siam wood EO | *Fokienia hodginsii* | Vietnam | 83.8±1.5 | 3 |
| 170 | Thuja EO | *Thuja plicata* | Central/East Europe | 0 | 7 |
| *Cyperaceae* | | | | | |
| 171 | Cypriol (Nagarmotha) EO | *Cyperus scariosus* R. Br. | India | 59.3±1.6 | 21 |
| *Dipterocarpaceae* | | | | | |
| 172 | Gurjun Balsam rectified (Copaene) | *Dipterocarpus spp* | Indonesia | 0 | 13 |
| *Ericaceae* | | | | | |
| 173 | Anthopogon EO | *Rhododendron anthopogon* | Nepal | 36 | 0 |
| 174 | Labrador tea EO | *Rhododendron groenlandicum* | Canada | 15 | 17 |
| 175 | Wintergreen EO | *Gaultheria procumbens* | Nepal | 0 | 26 |
| *Euphorbiaceae* | | | | | |
| 176 | Cascarilla bark EO | *Croton eluteria* (L.)  W. Wright  syn. *Clutia eluteria* L. | El Salvador | 30 | 2 |
| *Fabaceae* | | | | | |
| 177 | Broom absolute | *Spartium junceum* | Italy | 0 | 36 |
| 178 | Cabreuva red EO | *Myrocarpus fastigiatus* Allemao | Paraguay | 78.6±0.9 | 19 |
| 179 | Cassie absolute | *Acacia farnesiana* | Egypt | 0 | 7 |
| 180 | Copaiba balsam | *Copaifera officinalis* | Brazil | 10 | 8 |
| 181 | Fenugreek absolute | *Trigonella foenum graecum* | Egypt | 0 | 25 |
| 182 |  |  | India | 0 | 2 |
| 183 | Licorice hydroalcoholic extract | *Glycyrrhiza glabra* | Egypt | 0 | 23 |
| 184 | Mimosa absolute | *Acacia decurrens* var.*dealbata* | France | 38 | 5 |
| 185 | Paramela EO | *Adesmia Boronioides* Hook. F. | Argentina | 34 | 8 |
| 186 | Peru balsam | *Myroxylon balsamum* var*.*  *pereirae* | El Salvador | 64.4±2.2 | 36 |
| 187 | Peru balsam |  | Guatemala | 44 | 0 |
| 188 | Peru balsam raw |  | Guatemala | 20 | 15 |
| 189 | Peru resinoid |  | El Salvador | 57.9±1.5 | 9 |
| 190 | Tolu resinoid | *Myroxylon balsamum* (L.) Harms | Venezuela | 77.3±4.6 | 15 |
| 191 | Tonka bean absolute | *Dipteryx odorata* | Brazil | 0 | 21 |
| *Geraniaceae* | | | | | |
| 192 | Geranium EO | *Pelargonium graveolens* | China | 0 | 0 |
| 193 | Geranium EO |  | Greece | 27 | 0 |
| 194 | Geranium EO |  | Egypt | 29 | 23 |
| 195 | Geranium EO |  | Madagascar | 0 | 5 |
| 196 | Geranium EO |  | South Africa | 0 | 0 |
| 197 | Geranium Bourbon EO |  | Madagascar | 0 | 4 |
| 198 | Geranium absolute |  | Egypt | 27 | 22 |
| 199 | Geranium concrete |  |  | 30 | 13 |
| 200 | Geranium hydrolate absolute |  |  | 29 | 35 |
| *Grossulariaceae* | | | | | |
| 201 | Blackcurrant buds absolute | *Ribes nigrum* | France | 44 | 16 |
| *Iridaceae* | | | | | |
| 202 | Orris root absolute | *Iris pallida* | Italy | 13 | 0 |
| 203 | Orris root concentrate |  | France | 0 | 26 |
| *Lamiaceae* | | | | | |
| 204 | Basil cinnamon EO | *Ocimum basilicum ‘Cinnamon’* | Egypt | 7 | 3 |
| 205 | Basil holy EO | *Ocimum tenuiflorum* L.  syn. *Ocimum sanctum* L. | India | 19 | 7 |
| 206 | Basil lemon EO | *Ocimum x africanum* | Egypt | 32 | 0 |
| 207 | Basil lemon absolute |  |  | 25 | 42 |
| 208 | Basil lemon concrete |  |  | 26 | 30 |
| 209 | Basil oleoresin | *Ocimum basilicum* | India | 0 | 27 |
| 210 | Basil sweet (linalool) EO |  | Egypt | 23 | 0 |
| 211 | Basil sweet (linalool) EO |  | Greece | 22 | 8 |
| 212 | Basil sweet (linalool) EO |  | Thailand | 2 | 0 |
| 213 | Basil sweet (linalool) EO |  | Egypt | 34 | 33 |
| 214 | Basil sweet (linalool) EO |  |  | 30 | 31 |
| 215 | Basil tropical (estragol) EO |  | Egypt | 11 | 18 |
| 216 | Basil tropical (estragol) EO |  | India | 0 | 15 |
| 217 | Basil tropical (estragol) EO |  | Thailand | 3 | 8 |
| 218 | Basil tropical (estragol) absolute |  | Egypt | 17 | 29 |
| 219 | Basil tropical (estragol) concrete |  |  | 17 | 29 |
| 220 | Basil tropical (estragol) hydrolate absolute |  |  | 8 | 32 |
| 221 | Catnip (catmint) EO | *Nepeta cataria* | Canada | 21 | 0 |
| 222 | Hyssop EO | *Hyssopus officinalis* | Austria | 17 | 14 |
| 223 | Hyssop EO |  | Bulgaria | 3 | 18 |
| 224 | Lavandin EO | *Lavandula angustifolia* Mill. *× Lavandula latifolia* Medik. | France | 3 | 16 |
| 225 | Lavandin abrialis EO |  | France | 2 | 0 |
| 226 | Lavandin absolute |  | France | 0 | 8 |
| 227 | Lavandin grosso EO |  | France | 1 | 2 |
| 228 | Lavandin super EO |  | France | 0 | 0 |
| 229 | Lavandin EO |  | Greece | 17 | 3 |
| 230 | Lavender EO | *Lavandula angustifolia* Mill. | Australia | 16 | 9 |
| 231 | Lavender EO |  | France | 15 | 5 |
| 232 | Lavender EO |  | Greece | 21 | 8 |
| 233 | Lavender EO |  | Spain | 4 | 9 |
| 234 | Lavender fresh EO |  | Bulgaria | 2 | 13 |
| 235 | Lavender green absolute |  | France | 0 | 8 |
| 236 | Lavender organic EO |  | France | 0 | 7 |
| 237 | Lavender spike EO | *Lavandula latifolia* Medik. | France | 0 | 10 |
| 238 | Lavender spike absolute |  | France | 33 | 10 |
| 239 | Spanish wild marjoram EO | *Thymus mastichina* | Spain | 0 | 16 |
| 240 | Marjoram sweet EO | *Origanum majorana* | Greece | 15 | 0 |
| 241 | Marjoram sweet EO |  | Morocco | 0 | 12 |
| 242 | Marjoram sweet EO |  | Egypt | 3 | 17 |
| 243 | Marjoram sweet absolute |  |  | 39 | 30 |
| 244 | Lemon balm EO | *Melissa officinalis* | Bulgaria | 47 | 14 |
| 245 | Lemon balm EO |  | Greece | 43 | 0 |
| 246 | Mentha citrata EO | *Mentha aquatica* L. var. *citrata* (Ehrh.) Fresen.  syn. *Mentha citrata* Ehrh. | India | 0 | 0 |
| 247 | Mentha arvensis EO | *Mentha canadensis* L.  syn. *Mentha arvensis* var. *piperascens* Malinv. ex Holmes  syn. *Mentha arvensis* var. *glabrata* (Benth.) Fernald | China | 0 | 9 |
| 248 | Mentha arvensis EO |  | India | 0 | 12 |
| 249 | Mentha arvensis partially dementholized EO |  | India | 0 | 17 |
| 250 | Oregano EO | *Origanum vulgare*  L. subsp. *hirtum* (*Link*)  *letsw.* | Greece | 23 | 11 |
| 251 | Oregano EO, carvacrol 91.7% |  |  | 17 | 8 |
| 252 | Pennyroyal EO | *Mentha pulegium* | Morocco | 7 | 13 |
| 253 | Peppermint EO | *Mentha* x *piperita* | Australia | 16 | 1 |
| 254 | Peppermint EO |  | China | 0 | 22 |
| 255 | Peppermint Yakima EO |  | USA | 18 | 0 |
| 256 | Peppermint |  | Greece | 9 | 0 |
| 257 | Perilla EO | *Perilla frutescens* | China | 32 | 0 |
| 258 | Spearmint EO | *Mentha* spicata L.  syn. *Mentha viridis* L. var. *crispa* Benth. | China | 0 | 2 |
| 259 | Spearmint EO |  | Greece | 19 | 0 |
| 260 | Spearmint EO |  | Egypt | 13 | 12 |
| 261 | Origanum Spanish type EO | *Thymbra capitata*  (L.) Cav.  syn. *Thymus capitatus* (L.) Hoffmanns. & Link | Spain | 22 | 8 |
| 262 | Patchouli EO | *Pogostemon cablin* (Blanco) Benth.  syn. *Mentha cablin* Blanco | Indonesia | 63.5±2.0 | 12 |
| 263 | Patchouli DM Sulawesi Iron free EO |  | Indonesia | 11 | 10 |
| 264 | Patchouli light EO |  | Indonesia | 27 | 4 |
| 265 | Patchouli molecular distillation EO |  | Indonesia | 11 | 11 |
| 266 | Patchouli Super Dark EO |  | Indonesia | 45 | 26 |
| 267 | Rosemary EO | *Rosmarinus officinalis* | Australia | 19 | 11 |
| 268 | Rosemary EO |  | Spain | 0 | 11 |
| 269 | Rosemary EO |  | Greece | 37 | 4 |
| 270 | Rosemary EO |  | Egypt | 26 | 27 |
| 271 | Rosemary absolute |  |  | 46 | 29 |
| 272 | Sage clary EO | *Salvia sclarea* L.  syn. *Salvia sclarea* var. *turkestaniana* (Noter) Mottet | Egypt | 33 | 0 |
| 273 | Sage clary EO |  | Greece | 26 | 0 |
| 274 | Sage clary EO |  | Spain | 0 | 3 |
| 275 | Sage clary absolute |  | Egypt | 2 | 22 |
| 276 | Sage clary concrete |  |  | 0 | 7 |
| 277 | Sage Dalmatian EO | *Salvia officinalis* | Hungary | 0 | 7 |
| 278 | Sage Dalmatian EO | *Salvia officinalis* | Greece | 23 | 0 |
| 279 | Sage, Spanish type EO | *Salvia officinalis* subsp. *lavandulifolia* (Vahl) Gams  syn. *Salvia lavandulifolia* Vahl | Spain | 0 | 4 |
| 280 | Savory summer EO | *Satureja hortensis* | Egypt | 23 | 0 |
| 281 | Savory summer EO |  | Hungary | 19 | 7 |
| 282 | Savory winter EO | *Satureja montana* | Egypt | 15 | 0 |
| 283 | Thyme grey absolute | *Thymus zygis* | Spain | 13 | 8 |
| 284 | Thyme var thymol EO |  | Hungary | 16 | 5 |
| 285 | Thyme red EO | *Thymus vulgaris* | Spain | 10 | 8 |
| 286 | Thyme EO |  | Greece | 10 | 0 |
| 287 | Thyme red absolute |  | Spain | 0 | 13 |
| 288 | Thyme common absolute |  | Egypt | 37 | 47 |
| 289 | Thyme red oleoresin |  | Turkey | 0 | 28 |
| 290 | Thyme white EO |  | France | 2 | 4 |
| *Lauraceae* | | | | | |
| 291 | Camphor tree (camphor laurel) EO | *Cinnamomum camphora* (L.) J. Presl  syn. *Laurus camphora* L. | Japan | 0 | 0 |
| 292 | Cassia, Chinese type EO | *Cinnamomum cassia* (L.) J. Presl  syn. *Cinnamomum aromaticum* Nees | China | 35 | 8 |
| 293 | Cassia, Chinese type CO_2_ |  | Indonesia | 43 | 8 |
| 294 | Cinnamon bark, Sri Lanka type EO | *Cinnamomum verum*  J. Presl  syn. *Cinnamomum zeylanicum* Blume | Indonesia | 15 | 0 |
| 295 | Cinnamon leaf, Sri Lanka type EO |  | Indonesia | 0 | 0 |
| 296 | Ho (Shiu) wood EO | *Cinnamomum camphora* linaloliferum | China | 0 | 19 |
| 297 | Ishpingo EO | *Ocotea quixos* | Ecuador | 39 | 38 |
| 298 | Litsea cubeba EO | *Litsea cubeba* (Lour.) Pers. | China | 16 | 12 |
| 299 | Massoia bark EO | *Cryptocarya massoy* (Oken) Kosterm. | Indonesia | 6 | 4 |
| 300 | Ocotea cymbarum EO | *Ocotea cymbarum* | China | 11 | 0 |
| 301 | Ravintsara EO | *Cinnamomum camphora* | Madagascar | 11 | 30 |
| 302 | Rosewood, Brazilian type EO | *Aniba rosaeodora* Ducke  *Aniba parviflora* (Meissn.) Mez. | Brazil | 23 | 1 |
| 303 | Sassafras EO | *Sassafras albidum* | Taiwan | 14 | 0 |
| 304 | Sugandha kokila EO | *Cinnamomum glaucescens* | India | 17 | 1 |
| 305 | Sweet bay EO | *Laurus nobilis* | Spain | 0 | 11 |
| 306 | Sweet bay EO |  | Greece | 26 | 0 |
| 307 | Sweet bay oleoresin |  | Turkey | 15 | 13 |
| *Magnoliaceae* | | | | | |
| 308 | Magnolia champaca flower EO | *Michelia × alba* DC | Thailand | 39 | 0 |
| 309 | Magnolia champaca leaf EO |  | Thailand | 17 | 1 |
| *Malvaceae* | | | | | |
| 310 | Cocoa seed absolute | *Theobroma cacao* | Tanzania | 0 | 30 |
| 311 | Cocoa butter CO_2_ |  | Indonesia | 0 | 36 |
| *Meliaceae* | | | | | |
| 312 | Neem oil (exp.) | *Azadirachta indica* | India | 6 | 18 |
| *Myristicaceae* | | | | | |
| 313 | Mace EO | *Myristica fragrans* Houtt. | Indonesia | 0 | 0 |
| 314 | Nutmeg EO |  | India | 0 | 7 |
| 315 | Nutmeg EO |  | Indonesia | 0 | 11 |
| *Myrtaceae* | | | | | |
| 316 | Bay EO | *Pimenta racemosa* (Mill.) J.W. Moore | India | 34 | 1 |
| 317 | Big Badja gum EO | *Eucalyptus badjensis* | Australia | 10 | 0 |
| 318 | Blue mallee EO | *Eucalyptus polybractea* R. T. Baker | Australia | 4 | 17 |
| 319 | Cajeput EO | *Melaleuca leucadendra* | Indonesia | 0 | 2 |
| 320 | Clove bud CO_2_ | *Syzygium aromaticum* (L.) Merr. & L. M. Perry  syn. *Eugenia caryophyllus* (Spreng.) Bullock & S. G. Harrison | Indonesia | 25 | 14 |
| 321 | Clove bud EO |  | Madagascar | 40 | 20 |
| 322 | Clove leaf EO |  | Madagascar | 46 | 24 |
| 323 | Clove stem MD 85% EO |  | Indonesia | 25 | 14 |
| 324 | Clove stem dark 85% EO |  | Indonesia | 33 | 31 |
| 325 | Coastal tea tree EO | *Leptospermum laevigatum* | Australia | 44 | 35 |
| 326 | Eucalyptus citriodiora (lemon-scented gum) EO | *Corymbia citriodora* (Hook.) K.D. Hill & L.A.S. Johnson  syn. *Eucalyptus citriodora* Hook. | Brazil | 50.0±2.5 | 5 |
| 327 |  |  | Madagascar | 19 | 21 |
| 328 | Eucalyptus globulus EO | *Eucalyptus globulus* Labill. | Australia | 23 | 4 |
| 329 | Eucalyptus globulus EO |  | China | 0 | 5 |
| 330 | Eucalyptus kochii EO | *Eucalyptus kochii* | Australia | 0 | 1 |
| 331 | Eucalyptus radiata EO | *Eucalyptus radiata* Sieber ex DC | South Africa | 0 | 0 |
| 332 | Eucalyptus smithii EO | *Eucalyptus smithii* | South Africa | 0 | 10 |
| 333 | Kānuka EO | *Kunzea ericoides*  (A. Rich.) Joy Thomps.  syn. *Leptospermum ericoides* A. Rich. | New Zealand | 0 | 7 |
| 334 | Lemon Myrtle EO | *Backhousia citriodora* F. Muell. | Australia | 0 | 25 |
| 335 | Lemon scented tea tree EO | *Leptospermum petersonii* F. M. Bailey  syn. *Leptospermum citratum* Challinor, Cheel & Penfold | South Africa | 0 | 1 |
| 336 | Mānuka EO | *Leptospermum scoparium* J. R. Forst et G. Forst | Australia | 17 | 15 |
| 337 | Mānuka (MBTK 5+) EO |  | New Zealand | 16 | 5 |
| 338 | Mānuka (MBTK 20+) EO |  | New Zealand | 9 | 6 |
| 339 | Mānuka (MBTK 25+) EO |  | New Zealand | 7 | 33 |
| 340 | Melaleuca,  terpinen-4-ol type (tea tree) EO | *Melaleuca alternifolia* (Maiden & Betche) Cheel  syn. *Melaleuca linariifolia* Sm. | Australia | 0 | 0 |
| 341 | Melaleuca,  terpinen-4-ol type (tea tree) EO |  | South Africa | 0 | 5 |
| 342 | Melaleuca,  terpinen-4-ol type (tea tree) EO |  | China | 5 | 26 |
| 343 | Myrtle EO | *Myrtus communis* | Tunisia | 0 | 25 |
| 344 | Niaouli EO | *Melaleuca quinquenervia* (Cav.) S.T. Blake | Madagascar | 0 | 10 |
| 345 | Pimento berry EO | *Pimenta dioica* (L.) Merr. | Jamaica | 0 | 11 |
| 346 | Pimento leaf EO |  | Jamaica | 0 | 6 |
| 347 | Rosalina EO | *Melaleuca ericifolia* | Australia | 30 | 17 |
| 348 | Smoky tea tree EO | *Leptospermum glaucescens* | Australia | 37 | 31 |
| 349 | Sweet fern EO | *Comptonia peregrina* | Canada | 32 | 0 |
| 350 | Tick Bush EO | *Kunzea ambigua* (Sm.) Druce  syn. *Leptospermum ambiguum* Sm. | Australia | 16 | 4 |
| *Nymphaeaceae* | | | | | |
| 351 | Water lily absolute | *Nymphaea caerulea* | Sri Lanka | 0 | 13 |
| *Oleaceae* | | | | | |
| 352 | Jasmine EO | *Jasminum grandiflorum* | Egypt | 1 | 17 |
| 353 | Jasmine absolute |  | Egypt | 0 | 25 |
| 354 | Jasmine absolute |  | India | 0 | 16 |
| 355 | Jasmine concrete |  | Egypt | 0 | 28 |
| 356 | Olive leaf absolute | *Olea europea* | Egypt | 0 | 10 |
| 357 | Osmanthus absolute | *Osmanthus fragrans* | China | 0 | 7 |
| *Parmeliaceae* | | | | | |
| 358 | Oakmoss absolute | *Evernia Prunastri* | North Macedonia | 71.4±3.1 | 16 |
| 359 | Oakmoss resinoid |  | France | 28 | 11 |
| 360 | Treemoss absolute | *Pseudevernia furfuracea* | France | 12 | 18 |
| *Pinaceae* | | | | | |
| 361 | Black pine EO | *Pinus nigra* | Austria | 37 | 10 |
| 362 | Cedarwood atlas EO | *Cedrus atlantica* (Endl.) G. Manetti ex Carrière | Morocco | 54.4±4.6 | 1 |
| 363 | Cedarwood Himalaya EO | *Cedrus deodara* (Roxb. ex D. Don) G. Don | India | 5 | 15 |
| 364 | Fir needle Canadian EO | *Abies balsamea* (L.) Mill. | Canada | 3 | 21 |
| 365 | Fir needle Canadian EO |  | China | 0 | 0 |
| 366 | Fir balsam Canadian absolute |  | Canada | 37 | 0 |
| 367 | Fir balsam Canadian concrete |  |  | 35 | 0 |
| 368 | Fir Douglas | *Pseudotsuga menziesii* | Argentina | 23 | 14 |
| 369 | Fir needle Siberian EO | *Abies sibrica* Ledeb. | Russia | 21 | 26 |
| 370 | Hemlock, spruce EO | *Tsuga canadensis* L. Carriere | Canada | 0 | 7 |
| 371 | Jack pine twigs and leaves EO | *Pinus banksiana* |  | 14 | 9 |
| 372 | Jack pine wood EO |  |  | 8 | 7 |
| 373 | Larch tamarack | *Larix laricina* |  | 12 | 0 |
| 374 | Longleaf pine EO | *Pinus palustris* |  | 0 | 22 |
| 375 | Pine pumilio EO | *Pinus pumilio* | Germany | 31 | 0 |
| 376 | Scots pine EO | *Pinus sylvestris* | Bulgaria | 0 | 5 |
| 377 | Scots pine EO |  | Greece | 10 | 0 |
| 378 | Silver fir cones EO | *Abies alba* Mill. | Austria | 37 | 16 |
| 379 | Silver fir needles & twigs EO |  | Greece | 37 | 0 |
| 380 | Spruce black twigs and leaves EO | *Picea mariana* | Canada | 4 | 8 |
| 381 | Spruce black bark EO |  |  | 8 | 5 |
| 382 | Spruce black wood EO |  |  | 19 | 8 |
| 383 | Spruce white twigs and needles EO | *Picea glauca* |  | 14 | 0 |
| 384 | Spruce absolute | *Picea glauca/Picea mariana* |  | 52.8±1.8 | 0 |
| 385 | Swiss pine EO | *Pinus cembra* | Austria | 0 | 2 |
| 386 | Turpentine EO | *Pinus pinaster* Aiton | Austria | 40 | 14 |
| 387 | Venice turpentine I | *Pinus pinaster* Aiton | Austria | 56.3±2.8 | 30 |
| 388 | Yellow pine needles EO | *Pinus ponderosa* | Argentina | 24 | 7 |
| *Piperaceae* | | | | | |
| 389 | Betel leaf EO | *Piper betle* | India | 29 | 16 |
| 390 | Cubeb EO | *Piper cubeba* L.f. | Singapore | 43 | 0 |
| 391 | Cubeb CO_2_ |  | Indonesia | 47 | 24 |
| 392 | Pepper black oleoresin 40/20 | *Piper nigrum* | India | 16 | 46 |
| 393 | Pepper black EO |  | India | 0 | 5 |
| 394 | Pepper black EO |  | Madagascar | 24 | 17 |
| 395 | Pepper black CO_2_ |  | India | 0 | 36 |
| 396 | Pepper black CO_2_ |  | Indonesia | 38 | 26 |
| 397 | Spiked pepper (matico) EO | *Piper aduncum* | Ecuador | 18 | 9 |
| *Poaceae* | | | | | |
| 398 | Bran absolute | *Triticum vulgare* | France | 0 | 25 |
| 399 | Bran concrete |  | Morocco | 0 | 26 |
| 400 | Citronella, Sri Lanka type EO | *Cymbopogon nardus* (L.) W. Watson var. *lenabatu* Stapf. | Sri Lanka | 51.3±1.4 | 11 |
| 401 | Citronella, Java type EO | *Cymbopogon winterianus* Jowitt | Java Indonesia | 29 | 14 |
| 402 | Citronella organic, Java type EO |  | Paraguay | 31 | 16 |
| 403 | Citronella, Java type EO |  | China | 38 | 8 |
| 404 | Gingergrass EO | *Cymbopogon martinii* (Roxb.) W. Watson var. *sofia* | Indonesia | 21 | 33 |
| 405 | Hay absolute | *Lolium perenne* | France | 1 | 13 |
| 406 | Lemongrass West Indian EO | *Cymbopogon citratus* (DC.) Stapf. | China | 2 | 6 |
| 407 | Lemongrass EO | *Cymbopogon flexuosus* (Nees ex Steud.) W. Watson | India | 3 | 4 |
| 408 | Palmarosa EO | *Cymbopogon martini* (Roxb.) W. Watson var.  *motia* | Indonesia | 22 | 9 |
| 409 | Vetiver EO | *Chrysopogon zizanioides* (L.) Roberty  syn. *Vetiveria zizanioides* (L.) Nash | Brazil | 66.0±7.2 | 7 |
| 410 | Vetiver EO |  | Haiti | 74.2±4.3 | 0 |
| 411 | Vetiver EO |  | Java Indonesia | 65.9±4.4 | 35 |
| 412 | Vetiver EO |  | India | 45 | 16 |
| 413 | Vetiver CO_2_ |  | India | 0 | 3 |
| *Ranunculaceae* | | | | | |
| 414 | Black cumin seed oil (exp.) | *Nigella sativa* | India | 0 | 0 |
| 415 | Black cumin CO_2_ |  | India | 2 | 25 |
| *Rosaceae* | | | | | |
| 416 | Rose EO | *Rosa x damascena* Mill. | Bulgaria | 26 | 0 |
| 417 | Rose EO |  | Greece | 34 | 0 |
| 418 | Rose EO |  | Turkey | 25 | 0 |
| 419 | Rose absolute |  | Bulgaria | 43 | 0 |
| 420 | Rose absolute |  | Egypt | 31 | 9 |
| 421 | Rose absolute |  | Morocco | 26 | 0 |
| 422 | Rose cabbage EO | *Rosa centifolia* | Egypt | 6 | 17 |
| 423 | Rose cabbage absolute |  | Egypt | 0 | 13 |
| 424 | Rose cabbage leaves absolute |  | Egypt | 0 | 19 |
| 425 | Rose cabbage concrete |  | Egypt | 0 | 6 |
| 426 | Strawberry leaf absolute | *Fragaria x ananassa* | Egypt | 11 | 6 |
| *Rubiaceae* | | | | | |
| 427 | Coffee arabica absolute | *Coffea arabica* | Indonesia | 0 | 3 |
| 428 | Coffee arabica CO_2_ |  | India | 0 | 19 |
| 429 | Coffee Robusta CO_2_ | *Coffea canephora* | India | 0 | 1 |
| *Rutaceae* | | | | | |
| 430 | Amyris EO | *Amyris balsamifera* | Haiti | 57.9±4.9 | 16 |
| 431 | Bergamot EO | *Citrus bergamia* Risso & Poit.  syn. *Citrus aurantium* L. subsp. *bergamia* (Risso & Poit.) Wight & Arn. ex Engl. | Italy | 8 | 0 |
| 432 | Boronia absolute | *Boronia megastigma* | Australia | 24 | 33 |
| 433 | Boronia leaf absolute |  | Australia | 21 | 27 |
| 434 | Buchu leaf EO | *Agathosma betulina* (P.J. Bergius) Pillans | South Africa | 0 | 0 |
| 435 | Buchu leaf EO | *Agathosma crenulata* | South Africa | 4 | 0 |
| 436 | Citrus hystrix (kaffir lime) EO | *Citrus hystrix* DC.  syn. *Citrus torosa* Blanco | China | 0 | 0 |
| 437 | Clementine mandarin EO | *Citrus clementina* hort. ex Tanaca  syn. *Citrus deliciosa* Ten. *x Citrus sinensis* (L.) Osbeck | Spain | 0 | 16 |
| 438 | Grapefruit Pink EO | *Citrus x paradisi* Macfad. | USA | 0 | 5 |
| 439 | Grapefruit red EO |  | USA | 4 | 20 |
| 440 | Grapefruit White  EO |  | USA | 4 | 7 |
| 441 | Grapefruit White  EO |  | Greece | 6 | 0 |
| 442 | Lemon EO | *Citrus limon* (L.) Osbeck | Italy | 0 | 12 |
| 443 | Lemon EO |  | California USA | 0 | 0 |
| 444 | Lemon EO |  | Argentina | 0 | 0 |
| 445 | Lemon EO |  | Greece | 0 | 0 |
| 446 | Lemon EO |  | Spain | 3 | 12 |
| 447 | Lemon sfumatrice EO |  | Italy | 0 | 1 |
| 448 | Lime EO | *Citrus aurantifolia* (Christm.) Swingle | India | 0 | 0 |
| 449 | Lime distilled EO |  | Mexico | 0 | 9 |
| 450 | Lime expressed EO |  | Mexico | 0 | 25 |
| 451 | Mandarin EO | *Citrus reticulata* Blanco  syn. *Citrus nobilis* Andrews | Italy | 0 | 10 |
| 452 | Mandarin EO |  | Greece | 0 | 0 |
| 453 | Mandarin green EO |  | Brazil | 0 | 1 |
| 454 | Mandarin red EO |  | Argentina | 0 | 0 |
| 455 | Neroli EO | *Citrus aurantium* L.  syn. *Citrus amara* Link  syn. *Citrus* bigaradia Loisel  syn. *Citrus vulgaris* Risso | Morocco | 24 | 2 |
| 456 | Neroli EO |  | Egypt | 14 | 12 |
| 457 | Neroli BdN EO |  | Egypt | 15 | 0 |
| 458 | Orange bitter, blossom hydrolate absolute |  | Egypt | 27 | 22 |
| 459 | Orange bitter, blossom hydrolate absolute |  | Morocco | 29 | 22 |
| 460 | Orange bitter, distilled EO |  | Egypt | 0 | 0 |
| 461 | Orange bitter EO |  | Brazil | 0 | 9 |
| 462 | Orange bitter "Red" (mature) EO, cold pressed |  | Egypt | 23 | 20 |
| 463 | Orange bitter "Green" (immature) EO, cold pressed |  | Egypt | 9 | 24 |
| 464 | Orange blood EO | *Citrus sinensis* (L.) Osbeck | Italy | 0 | 5 |
| 465 | Orange flower absolute | *Citrus aurantium* L.  syn. *Citrus amara* Link  syn. *Citrus* bigaradia Loisel  syn. *Citrus vulgaris* Risso | Tunisia | 13 | 5 |
| 466 | Orange flower concrete |  | Morocco | 47 | 35 |
| 467 | Orange sweet (high aldehyde) EO | *Citrus sinensis* (L.) Osbeck | Brazil | 0 | 5 |
| 468 | Orange sweet EO |  | Brazil | 3 | 14 |
| 469 | Orange sweet EO |  | USA | 0 | 20 |
| 470 | Orange sweet EO |  | Valencia Spain | 0 | 7 |
| 471 | Orange sweet EO |  | Greece | 12 | 4 |
| 472 | Orange sweet 5-fold EO |  | Brazil | 0 | 10 |
| 473 | Petitgrain bigarade EO | *Citrus aurantium* L.  syn. *Citrus amara* Link  syn. *Citrus* bigaradia Loisel  syn. *Citrus vulgaris* Risso | Egypt | 15 | 9 |
| 474 | Petitgrain bigarade EO |  | Greece | 6 | 0 |
| 475 | Petitgrain bigarade *sur fleurs* EO |  | Egypt | 0 | 14 |
| 476 | Petitgrain bigarade EO |  | Paraguay | 5 | 15 |
| 477 | Petitgrain bigarade EO |  | Tunisia | 0 | 4 |
| 478 | Petitgrain bigarade absolute |  | Egypt | 8 | 33 |
| 479 | Petitgrain citrus hystrix (kaffir lime) EO | *Citrus hystrix* DC.  syn. *Citrus torosa* Blanco | China | 50 | 0 |
| 480 | Petitgrain key lime EO | *Citrus aurantifolia* (Christm.) Swingle | Egypt | 34 | 8 |
| 481 | Petitgrain lemon EO | *Citrus limon* (L.) Osbeck | Spain | 0 | 10 |
| 482 | Petitgrain mandarin EO | *Citrus reticulata* Blanco  syn. *Citrus nobilis* Andrews | Egypt, Spain | 85.3±6.2 | 100 |
| 483 | Petitgrain mandarin light fraction EO |  | Egypt | 69.7±4.5 | 100 |
| 484 | Petitgrain mandarin heavy fraction EO |  | Egypt | 87.3±4.4 | 100 |
| 485 | Rue EO | *Ruta graveolens* | Spain | 0 | 20 |
| 486 | Sichuan pepper (Limonella) EO | *Zanthoxylum limonella* Alston  syn. *Zanthoxylum rhetsa* (Roxb.) DC. | Mexico | 19 | 3 |
| 487 | Tangerine EO | *Citrus tangerina* | USA | 0 | 4 |
| 488 | Tangerine EO |  | Spain | 0 | 12 |
| 489 | Winged prickly ash EO | *Zanthoxylum armatum* | India | 2 | 13 |
| 490 | Yuzu EO | *Citrus junos* | Japan | 8 | 9 |
| *Salicaceae* | | | | | |
| 491 | Balsam poplar buds EO | *Populus balsamifera* | Canada | 41 | 6 |
| *Santalaceae* | | | | | |
| 492 | Australian sandalwood EO | *Santalum spicatum* (R.Br.) A. DC  syn. *Eucarya spicata* (R.Br.) Sprag et Summ. | Australia | 67.0±5.0 | 25 |
| 493 | Sandalwood EO | *Santalum album* | India | 1 | 7 |
| 494 | Sandalwood EO | *Santalum austro-caledonicum* | New Caledonia | 70.9±3.3 | 4 |
| *Schisandraceae* | | | | | |
| 495 | Star anise, Chinese type EO | *Illicium verum* Hook. f. | China | 8 | 0 |
| 496 | Star anise, Chinese type CO_2_ |  | India | 0 | 7 |
| *Scrophulariaceae* | | | | | |
| 497 | False sandalwood (buddawood) EO | *Eremophila mitchellii* Benth. | Australia | 40 | 17 |
| *Solanaceae* | | | | | |
| 498 | Capsicum CO_2_ | *Capsicum annuum* var. *annuum* | India | 16 | 28 |
| 499 | Capsicum oleoresin 1.000.000 SHU |  | India | 0 | 16 |
| 500 | Fabiana EO | *Fabiana imbricata* | Argentina | 33 | 14 |
| 501 | Paprika oleoresin 40.000 CU | *Capsicum annuum* | India | 3 | 49 |
| 502 | Paprika oleoresin 60.000 CU |  | India | 12 | 55.5±2.8 |
| 503 | Paprika oleoresin 80.000 CU |  | India | 19 | 45 |
| 504 | Tobacco absolute | *Nicotiana tabacum* | France | 0 | 20 |
| 505 | Tomato leaf abs | *Lycopersicum esculentum* | Egypt | 0 | 19 |
| *Styracaceae* | | | | | |
| 506 | Benzoin Siam resinoid | *Styrax tonkinensis* | Laos | 99.7±0.4 | 17 |
| 507 | Benzoin Sumatra resinoid | *Styrax benzoin* | Indonesia | 72.9±2.5 | 9 |
| *Tropaeolaceae* | | | | | |
| 508 | Nasturtium absolute | *Tropaeolum nasturtium* | Egypt | 67.8±2.0 | 25 |
| *Urticaceae* | | | | | |
| 509 | Nettle abs | *Urtica urens* | Egypt | 67.1±5.8 | 25 |
| *Valerianaceae* | | | | | |
| 510 | Valerian EO | *Valeriana officinalis* | China | 24 | 0 |
| *Verbenaceae* | | | | | |
| 511 | Lantana EO | *Lantana camara* | Egypt | 34 | 32 |
| 512 | Lemon verbena EO | *Aloysia citriodora* Palau  syn. *Aloysia triphylla* (L'Hér.) Britton  syn. *Lippia triphylla* (L'Hér.) Kuntze  syn. *Verbena triphylla* L'Hér.  syn. *Lippia citriodora* (Palau) Kunth | Morocco | 6 | 18 |
| *Violaceae* | | | | | |
| 513 | Violet leaf absolute | *Viola odorata* | France | 29 | 26 |
| 514 | Violet leaf absolute |  | Egypt | 56.2±9.5 | 23 |
| 515 | Violet leaf concrete |  |  | 49 | 20 |
| *Vitaceae* | | | | | |
| 516 | Cognac green EO | *Vitis vinifera* | Austria | 0 | 10 |
| 517 | Cognac white EO |  | USA | 0 | 0 |
| *Winteraceae* | | | | | |
| 518 | Tasmanian pepperberry absolute | *Tasmannia lanceolata* | Australia | 62.3±3.5 | 16 |
| 519 | Tasmanian pepperberry extract |  | Australia | 64.7±2.5 | 16 |
| *Zingiberaceae* | | | | | |
| 520 | Cardamom EO | *Elettaria cardamomum* (L.) Maton | Guatemala | 25 | 12 |
| 521 | Cardamom EO |  | India | 2 | 0 |
| 522 | Cardamom EO |  | Indonesia | 12 | 22 |
| 523 | Cardamom green CO_2_ |  | India | 0 | 14 |
| 524 | Ginger CO_2_ | *Zingiber officinale* Roscoe | India | 14 | 15 |
| 525 | Ginger CO_2_ |  | Indonesia | 38 | 0 |
| 526 | Ginger CO_2_ |  | China | 22 | 2 |
| 527 | Ginger EO |  | India | 39 | 35 |
| 528 | Ginger EO |  | Jamaica | 20 | 10 |
| 529 | Ginger EO |  | China | 23 | 16 |
| 530 | Ginger EO |  | Nigeria | 21 | 16 |
| 531 | Ginger fresh EO |  | Madagascar | 39 | 30 |
| 532 | Lesser galangal EO | *Alpinia officinarum* | China | 0 | 16 |
| 533 | Phlai EO | *Zingiber montanum* (J. König) Theilade  syn. *Zingiber cassumunar* Roxb. | Thailand | 4 | 6 |
| 534 | Turmeric EO | *Curcuma longa* | China | 63.8±2.1 | 6 |
| 535 | Turmeric CO_2_ |  | India | 33 | 5 |
| 536 | Turmeric CO_2_ |  | Indonesia | 28 | 10 |
| 537 | Turmeric oleoresin |  | India | 89.0±0.7 | 64.3±1.6 |
| *Zygophyllaceae* | | | | | |
| 538 | Guaiacwood EO | *Bulnesia sarmientoi* | Paraguay | 72.0±5.1 | 15 |

# **Inhibitory activity of plant extracts against SARS-CoV-1 proteases**

Table S2. Inhibitory activity of plant extracts and their constituents on the key SARS-CoV proteases.

| No | Material | SARS-CoV M^pro^ IC_50_ | SARS-CoV  PL^pro^ IC_50_ | Ref |
| --- | --- | --- | --- | --- |
| 1 | 3-Isotheaflavin-3-gallate | 7 µM | - | (Chen, Lin, Huang, Chen, Hsieh, Liang, et al., 2005) |
| 2 | Tannic acid | 3 µM | - |  |
| 3 | *Houttuynia cordata* aq extract | ~1000 µg/mL | - | (Lau, Lee, Koon, Cheung, Lau, Ho, et al., 2008) |
| 4 | *Psoralea corylifolia* seeds EtOH extract | - | 15 µg/mL | (Kim, Seo, Curtis-Long, Oh, Oh, Cho, et al., 2014) |
| 5 | *Rheum palmatum* EtOH extract | 38.09 µg/mL | - | (Luo, Su, Gong, Qin, Liu, Li, et al., 2009) |

# **Antiviral assay of selected F&F materials**

Table S3. Antiviral properties of the most active natural SARS-CoV-2 proteases inhibitors.

| **Natural material** | **SARS-CoV-2 EC_50_ [µg/mL]** | **Toxicity CC_50_ [µg/mL]** |
| --- | --- | --- |
| **Essential oils** | | |
| Angelica root India EO | >100 | 47.3±7.1 |
| Blue cypress EO | >100 | 28.5±1.1 |
| Cabreuva Red EO | >100 | 17.2±2.1 |
| Cade crude EO | >100 | 71.2±19.7 |
| Guaiacwood EO | >100 | 25.4±3.9 |
| Koron EOs* | >100 | >100 |
| Lovage root EO | >100 | 34.2±2.6 |
| Petitgrain mandarin EO | >100 | >100 |
| DMA | >100 µM | >100 |
| *Sandalwood spicatum* EO | >100 | 27.5±0.1 |
| Siam wood EO | >100 | 17.6±0.8 |
| **Extracts** | | |
| Amber solid | >100 | >100 |
| Benzoin Siam resinoid | 78.8±3.3 | >100 |
| Benzoin Sumatra resinoid | 31.5±2.4 | 85.5±1.9 |
| Galbanum resinoid | >100 | 9.2±0.1 |
| Nasturtium absolute | >100 | 58.2±5.5 |
| Nettle absolute | 94.9±4.1 | 94.9±4.1 |
| Oakmoss absolute | >100 | 83.4±1.8 |
| Paprika oleoresin 60 000 CU | >50 | >100 |
| Rocket absolute | >100 | 28.4±1.3 |
| Spinach absolute | >100 | 88.3±1.7 |
| Storax resinoid | >100 | 85.6±1.8 |
| *Tasmania lanceolata* extract | >100 | 28.3±0.6 |
| Tolu resinoid | >100 | >100 |
| Turmeric oleoresin | >100 | 28.1±0.1 |

*Mixture of essential oils used by the indigenous people of the Amazon to fight COVID-19.

# **Anti-SARS-CoV-1 activity of essential oils and plant extracts**

Table S4. The anti-SARS-CoV activity of plant extracts and their constituents.

| No | Material | SARS-CoV IC_50_ | Ref |
| --- | --- | --- | --- |
| 1 | *Cinnamomi cortex* (*Cinnamomum verum*, cinnamon bark) aq extract (CCE) | 43.1 µg/mL | (Zhuang, Jiang, Suzuki, Li, Xiao, Tanaka, et al., 2009) |
| 2 | *Cinnamomi cortex* (*Cinnamomum verum*, cinnamon bark) hydroalcoholic extract (CC) | 10.7 µg/mL |  |
| 3 | Butanol fraction of CC | 7.8 µg/mL |  |
| 4 | Aqueous fraction of CC | 39.7 µg/mL |  |
| 5 | *Caryophylli flos* (*Syzygium aromaticum*, clove buds) aq extract | 50.1 µg/mL |  |
| 6 | tetra-*O*-galloyl-β-D-glucose | 4.5 µM | (Yi, Li, Yuan, Qu, Chen, Wang, et al., 2004) |
| 7 | Luteolin | 10.6 µM |  |
| 8 | Glycyrrhizin | >607.6 µM |  |
| 9 | Glycyrrhizin | 300 µg/mL | (Cinatl, Morgenstern, Bauer, Chandra, Rabenau, & Doerr, 2003) |
| 10 | *Laurus nobilis* EO | 120 µg/mL | (Loizzo, Saab, Tundis, Statti, Menichini, Lampronti, et al., 2008) |
| 11 | *Juniperus oxycedrus* ssp. *oxycedrus* EO | 270 µg/mL |  |
| 12 | *Thuja orientalis* EO | 130 µg/mL |  |
| 13 | *Cupressus sempervirens* ssp. *pyramidalis* EO | 700 µg/mL |  |
| 14 | *Pistacia palaestina* EO | >1000 µg/mL |  |
| 15 | *Salvia officinalis* EO | 870 µg/mL |  |
| 16 | *Satureja thymbra* EO | No activity |  |

# **IC_50­_ graphs for PL^pro^**

Fig. S2.1. IC_50_ graphs (PL^pro^) for petitgrain mandarin (PM) essential oil and dimethyl anthranilate (DMA)

Fig. S2.2. IC_50_ graph (PL^pro^) for turmeric oleoresin.

# **IC_50­_ graphs for M^pro^**

| Essential Oils | |
| --- | --- |
|   Fig. S3.1. IC_50_ graph (M^pro^) for lovage root EO. |   Fig. S3.2. IC_50_ graph (M^pro^) for Siam wood EO. |
|   Fig. S3.3. IC_50_ graph (M^pro^) for blue cypress EO. |   Fig. S3.4. IC_50_ graph (M^pro^) for guaiacwood EO. |
| Extracts | |
|   Fig. S3.5. IC_50_ graph (M^pro^) for benzoin Siam resinoid. |   Fig. S3.6. IC_50_ graph (M^pro^) for benzoin Sumatra resinoid. |
|   Fig. S3.7. IC_50_ graph (M^pro^) for galbanum resinoid. |   Fig. S3.8. IC_50_ graph (M^pro^) for storax resinoid. |
|   Fig. S3.9. IC_50_ graph (M^pro^) for turmeric oleoresin. |   Fig. S3.10. IC_50_ graph (M^pro^) for labdanum gum refined. |
|   Fig. S3.11. IC_50_ graph (M^pro^) for nasturtium absolute. |   Fig. S3.12. IC_50_ graph (M^pro^) for rocket absolute. |
| Fig. S3.13. IC_50_ graph (M^pro^) for *Tasmania lanceolata* extract. | Fig. S3.14. IC_50_ graph (M^pro^) for rocket concrete. |

# **References**

Chen, C.-N., Lin, C. P. C., Huang, K.-K., Chen, W.-C., Hsieh, H.-P., Liang, P.-H., & Hsu, J. T. A. (2005). Inhibition of SARS-CoV 3C-like Protease Activity by Theaflavin-3,3'-digallate (TF3). *Evidence based complementary and alternative medicine, 2*(2), 209-215.

Cinatl, J., Morgenstern, B., Bauer, G., Chandra, P., Rabenau, H., & Doerr, H. W. (2003). Glycyrrhizin, an active component of liquorice roots, and replication of SARS-associated coronavirus. *The Lancet, 361*(9374), 2045-2046.

Kim, D. W., Seo, K. H., Curtis-Long, M. J., Oh, K. Y., Oh, J.-W., Cho, J. K., Lee, K. H., & Park, K. H. (2014). Phenolic phytochemical displaying SARS-CoV papain-like protease inhibition from the seeds of *Psoralea corylifolia*. *Journal of Enzyme Inhibition and Medicinal Chemistry, 29*(1), 59-63.

Lau, K.-M., Lee, K.-M., Koon, C.-M., Cheung, C. S.-F., Lau, C.-P., Ho, H.-M., Lee, M. Y.-H., Au, S. W.-N., Cheng, C. H.-K., Lau, C. B.-S., Tsui, S. K.-W., Wan, D. C.-C., Waye, M. M.-Y., Wong, K.-B., Wong, C.-K., Lam, C. W.-K., Leung, P.-C., & Fung, K.-P. (2008). Immunomodulatory and anti-SARS activities of Houttuynia cordata. *Journal of Ethnopharmacology, 118*(1), 79-85.

Loizzo, M. R., Saab, A. M., Tundis, R., Statti, G. A., Menichini, F., Lampronti, I., Gambari, R., Cinatl, J., & Doerr, H. W. (2008). Phytochemical Analysis and *in vitro* Antiviral Activities of the Essential Oils of Seven Lebanon Species. *Chemistry & Biodiversity, 5*(3), 461-470.

Luo, W., Su, X., Gong, S., Qin, Y., Liu, W., Li, J., Yu, H., & Xu, Q. (2009). Anti-SARS coronavirus 3C-like protease effects of *Rheum palmatum* L. extracts. *Bioscience trends, 3*(4), 124-126.

Yi, L., Li, Z., Yuan, K., Qu, X., Chen, J., Wang, G., Zhang, H., Luo, H., Zhu, L., Jiang, P., Chen, L., Shen, Y., Luo, M., Zuo, G., Hu, J., Duan, D., Nie, Y., Shi, X., Wang, W., Han, Y., Li, T., Liu, Y., Ding, M., Deng, H., & Xu, X. (2004). Small Molecules Blocking the Entry of Severe Acute Respiratory Syndrome Coronavirus into Host Cells. *Journal of Virology, 78*(20), 11334.

Zhuang, M., Jiang, H., Suzuki, Y., Li, X., Xiao, P., Tanaka, T., Ling, H., Yang, B., Saitoh, H., Zhang, L., Qin, C., Sugamura, K., & Hattori, T. (2009). Procyanidins and butanol extract of *Cinnamomi Cortex* inhibit SARS-CoV infection. *Antiviral Research, 82*(1), 73-81.
